# A Vascularized Human Organ Chip Reveals SARS-CoV-2 Susceptibility in Developmentally Guided Tissue Maturation

**DOI:** 10.1007/s12195-025-00851-4

**Published:** 2025-07-22

**Authors:** Titilola D. Kalejaiye, Rohan Bhattacharya, Samira Musah

**Affiliations:** 1 Department of Biomedical Engineering, Pratt School of Engineering, Duke University, Durham, NC USA; 2 Center for Biomolecular and Tissue Engineering, Duke University, Durham, NC USA; 3 Division of Nephrology, Department of Medicine, Duke University School of Medicine, Durham, NC USA; 4 Developmental and Stem Cell Biology Program, Duke University, Durham, NC USA; 5 Department of Cell Biology, Duke University, Durham, NC USA

**Keywords:** Human disease model, Microphysiological system, Stem cell differentiation, SARS-CoV-2, Viral infection, Kidney disease, Organs-on-chips

## Abstract

**Purpose:**

Stem cell-derived models offer traceable cell sources for studying tissue development and disease mechanisms. However, many such models have inherently immature or fetal-like phenotypes, limiting their relevance for mechanistic studies of specialized adult tissues. Clinical observations suggest a potential link between epithelial cells and their transit-amplifying progenitors in disease onset and viral tropism, but experimental validation is needed. This study aimed to develop mature visceral epithelial cells (podocytes) from human induced pluripotent stem (iPS) cells using a developmental approach and model severe acute respiratory syndrome coronavirus 2 (SARS-CoV-2) infection in a vascularized microfluidic kidney-on-a-chip platform exhibiting in vivo-like tissue structure and function.

**Methods:**

Mature podocytes and vascular endothelial cells were differentiated from patient-specific human iPS cells by transitioning through distinct lineages that mimic human development. A personalized vascularized microphysiological platform containing the stem cell-derived kidney cells was engineered to model glomerular tissue and the kidney’s blood filtration barrier. SARS-CoV-2 entry mechanisms and cell lineage marker expression were assessed at the transcriptome and proteome levels in the developing and mature cells and tissues.

**Results:**

The vascularized kidney-on-a-chip model revealed that susceptibility to SARS-CoV-2 particles was significantly higher in mature glomerular epithelium compared to less specialized derivatives and progenitor cells. The infection with SARS-CoV-2 also induced altered expression of cell lineage markers, with mature podocytes exhibiting distinct transcriptional responses linked to viral interacting epitopes and entry pathways.

**Conclusions:**

This study underscores the importance of using developmentally appropriate preclinical models to investigate disease mechanisms and potential therapeutic responses. These findings highlight the maturation-dependent susceptibility of specialized epithelial cells to viral infections, providing insights into organ-specific disease mechanisms and potential therapeutic strategies. These insights reinforce the need to refine preclinical model systems to closely align with human physiology and ensure the translational relevance of biomedical research.

**Supplementary Information:**

The online version contains supplementary material available at 10.1007/s12195-025-00851-4.

## Introduction

The coronavirus disease 2019 (COVID-19) is caused by the severe acute respiratory syndrome coronavirus 2 (SARS-CoV-2). While the availability of effective vaccines has improved outcomes for affected individuals and the general population, the COVID-19 pandemic has had global consequences and long-term economic and health impacts. Since the initial report of the disease in 2019, the SARS-CoV-2 virus has spread globally and by 2024 has infected more than 775 million people and caused about 7.0 million deaths. The most severe forms of the disease often occur in the elderly and individuals with underlying predispositions or comorbidities including hypertension, obesity, chronic kidney disease and diabetes [1, 2]. Although therapeutic antibodies are now available, SARS-CoV-2 variants such as Omicron and its sub-lineages continue to mutate and evade monoclonal antibodies and antibodies elicited by vaccination [3]. Understanding the cellular and molecular mechanisms of SARS-CoV-2 infection and COVID-19 disease progression is important for the development of effective antiviral therapies and preventative measures to address current and future coronavirus infections and diseases.

Coronaviruses are viruses of zoonotic origins, which cause severe pneumonia in humans and produce upper and lower respiratory tract infections in adults and children [4]. SARS-CoV-2 initially targets the respiratory epithelium of the nasal cavity, followed by wide-range multi-organ tropism including brain, kidney, liver, heart, and eyes [5]. In the kidney and other organs, infections by SARS-CoV-2 indicate multiple cellular vulnerabilities. These susceptibilities suggests involvement of several cell surface molecules for viral entry or cellular uptake [5] indicating possible participation of multiple cell types, including progenitor cells [6, 7]. It is therefore important to study cell-type specific responses and mechanisms in the kidney because acute kidney injury (AKI) and other renal complications are common (38% to 65%) in patients clinically diagnosed with severe COVID-19 disease [8–10].

Due to ethical concerns and limited access to human biological samples, efforts to study mammalian tissue and organ biology in vivo is extremely limited. However, advances in human stem cell-derived models have provided powerful alternatives and help address these limitations. For example, stem cell-derived models of several tissues and organs have been used to study the mode and mechanisms of SARS-CoV-2 infections [11–13] and pathogenesis including COVID-19 multi-organ dysfunction [5, 14–16]. We and others revealed that SARS-CoV-2 can utilize multiple classes of cell surface receptors, including Angiotensin-Converting Enzyme 2 (ACE2), Basigin (BSG, also known as CD147), CD209 (also known as DC-SIGN), and Neuropilin-1 (NRPI) to directly infect host cells [9, 12, 17, 18]. We also demonstrated that SARS-CoV-2 could infect and replicate in the specialized human kidney glomerular epithelial cells (podocytes) whereby the infected cells serve as reservoirs for viral replication and secondary infections, causing changes in gene expression and cell phenotype [12]. Additionally, we discovered that human kidney podocytes express multiple SARS-CoV-2 receptors actively employed for viral uptake, including ACE2 and BSG/CD147. Together, these prior discoveries confirm that kidney glomerular cells are extra-pulmonary targets of SARS-CoV-2 infection and pathogenesis, laying the foundation for subsequent investigations.

While several studies have assessed SARS-CoV-2 binding and uptake in a number of cell types and organoids [19–21] many of the in vitro models consist of immature or fetal-like cell types that cannot accurately represent the biological responses of specialized organ-specific cell types or adult tissues [22]. Thus, it remains less clear how progenitor and transit-amplifying cells contribute to SARS-CoV-2 infections and mechanisms, and how the responses compare to infections in mature and functional cell types such as those found in postnatal and adult tissues. In this study, using the S-pseudotyped virus, we investigated SARS-CoV-2 permissiveness and infection profiles in cells at different stages of tissue development, including stem cells and their lineage restricted derivatives up to the mature kidney glomerular podocyte. S-pseudotyped viruses are viral particles engineered to display the spike protein of SARS-CoV-2 on the surface but lack replicative ability. To examine tissue-level uptake of the virus, we employed a vascularized microfluidic organ chip (organ-on-a-chip) model of the human kidney glomerulus engineered from isogenic stem cell-derived epithelium and endothelium. We uncovered distinct molecular signatures and differential SARS-CoV-2 infectivity in mature and immature or transit-amplifying cell lineages.

## Results

### Expression Levels of Spike-Interacting Factors Vary Dynamically with Cell Developmental State

**Fig. 1:**
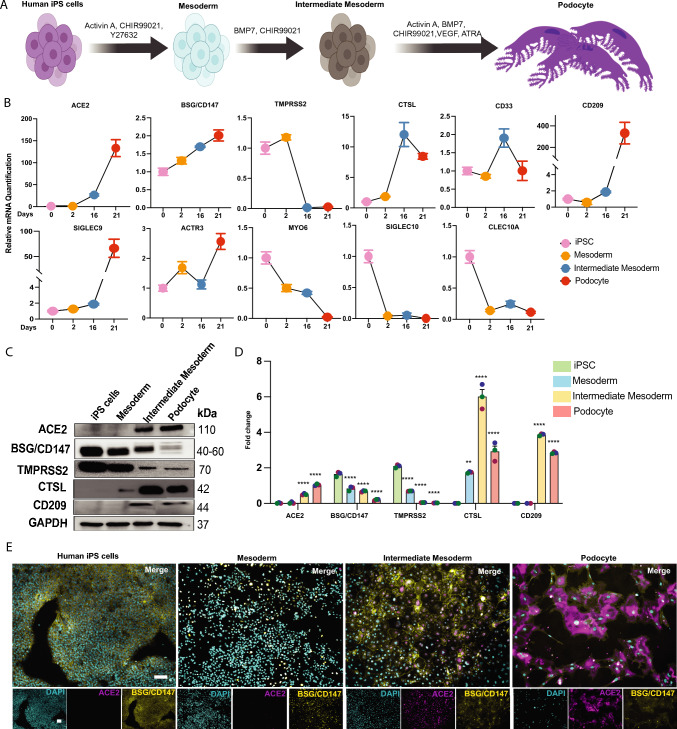
Dynamic expression levels of spike-interacting factors identified in pluripotent stem cells and their differentiated derivatives at varying cell maturity. **A** Schematic overview of the protocol for the differentiation of mature podocytes from human iPS cells; adapted from Musah et al. [23]. **B** Line plot of qPCR quantification of human spike-associated genes and the viral processing factors Transmembrane Serine Protease 2 (TMPRSS2) and cathepsin L (CTSL) in the cell lineages (normalized to human iPS cell group). SIGLEC9, Sialic acid-binding Ig-like lectin 9; CLEC10A, C-type lectin domain family 10 member A; CD33, Myeloid cell surface antigen CD33; ACE2, Angiotensin Converting Enzyme 2; BSG/CD147, Basigin/CD147 molecule; CD209, CD209 Antigen; MYO6, Unconventional myosin-VI; SIGLEC10, Sialic acid-binding Ig-like lectin 10; ACTR3, Actin-related protein 3. Error bars indicate ± SEM (standard error of the mean). Representative **C** western blot with **D** densitometric quantification of three biological replicates from cell lysates of all cell lineages, including iPS cells, mesoderm, IM cells, and podocytes showing ACE2, BSG/CD147, CD209, TMPRSS2 and CTSL proteins in the different cell lineages; GAPDH was used as a loading control. One-way analysis of variance (ANOVA) with Sidak’s multiple comparison test was used to determine statistical significance. Only p-values of 0.05 or lower were considered statistically significant. p > 0.05 [ns, not significant], p < 0.05 [*], p < 0.01 [**], p < 0.001 [***], p < 0.0001 [****]). **E** Immunofluorescence analysis of ACE2 (magenta) and BSG/CD147 (yellow) expression in the different cell lineages counterstained with DAPI (cyan). Scale bar: 100 µm. Figure **A** was created with Biorender^TM^

We recently discovered that mature human kidney glomerular podocytes express high levels of SARS-CoV-2 host factors (receptors and processing enzymes) and employ multiple receptors (in addition to ACE2) to facilitate infection by the virus [12]. Because numerous in vitro models for studying SARS-CoV-2 renal tropism contain cells that are developmentally immature, we wondered if kidney progenitors or developing cells express spike factors and whether the abundance of these factors changes and affects viral infectivity depending on the cell’s functional or developmental stage. To address this question, we differentiated human iPS cells to generate kidney lineage progenitors and mature glomerular podocytes (Fig. 1A) using our established protocol [23–25]. Because SARS-CoV-2 infection requires expression of entry and processing factors to mount virulence in the host cell, we analyzed and compared the expression of spike-interacting factors between the different kidney lineage cell types.

We initially compared the undifferentiated human iPS cells and their derivatives [mesoderm and intermediate mesoderm (IM)] for the expression of SARS-CoV-2 entry and processing factors [9, 12, 17, 18]. We quantified the basal mRNA expression levels of some of the spike associated genes in the different cell lineages and observed a substantial difference in the expression of the genes as the cells transitioned from the undifferentiated state (human iPS cells) to progenitor (mesoderm and IM) and terminally differentiated podocyte phenotypes (Figs. 1B and S1A). Compared to undifferentiated iPS cells, there were considerable differences in gene expression in IM cells and podocytes. For instance, ACE2 expression increased as cells matured from the IM stage to podocytes (the most mature cell type). There was a steady increase in BSG/CD147 mRNA expression from human iPS cells to podocytes. We also observed a significant decrease in Transmembrane Serine Protease 2 (TMPRSS2) mRNA expression in IM and podocytes, approaching null levels. We also examined mRNA levels of CD209, another protein found to permit entry of the virus [17]. CD209 mRNA level increases exponentially as the cells develop from the IM to the podocyte state. It is worth noting that cathepsin L (CTSL) mRNA levels increased significantly from mesoderm to IM and remained high in podocytes—signifying a possible endo-lysosomal entry pathway for the virus. The mRNA levels of additional genes such as Actin-related protein 3 (ACTR3), Sialic acid-binding Ig-like lectin 9 (SIGLEC9), Sialic acid-binding Ig-like lectin 10 (SIGLEC10), C-type lectin domain family 10 member A (CLEC10A), and Unconventional myosin-VI (MYO6) varied between the different cell lineages (Fig. 1B). SIGLECs are sialic-acid binding immunoglobulin-like lectins [26] often found in macrophages and activated T cells [27] and have been shown to bind specific spike proteins displayed on SARS-CoV-2 [28]. We observed that SIGLEC9 mRNA levels increased from human iPS cells to other cell derivatives, with an exponential upregulation in terminally differentiated podocytes; inverse relationships were observed for SIGLEC10 expression levels in the cell lineages. ACTR3, a member of the ARP2/3 complex (actin-related protein) essential for initial attachment and endocytosis of the virus [29, 30], was most highly expressed in podocytes when compared to human iPS cells and other progenitors. Additionally, CLEC10A, a glycan-dependent binding partner of SARS-CoV-2 spike [31], and MYO6 decreased in expression as the cells progressed from undifferentiated to differentiated lineages.

The protein level expression of uptake (ACE2, BSG/CD147, CD209) and two processing (TMPRSS2 and CTSL) factors were evaluated in all four cell lineages (undifferentiated human iPS cells, mesoderm, IM, and mature podocytes). Western blot analysis and quantification confirmed extremely low to no expression of ACE2 in undifferentiated human iPS cells and mesoderm cells and markedly high levels in IM cells and podocytes (Fig. 1C and D), consistent with the gene expression data in Fig. 1B. The western blot results demonstrated that podocytes express the highest level of ACE2 protein, followed by IM cells. Additionally, TMPRSS2 expression was highest in iPS cells, followed by mesoderm, and was detected at very low levels in IM cells and podocytes. Notably, the protein level expression of BSG/CD147 was highest in human iPS cells and decreased as the cells differentiated and matured, although the mRNA expression (Fig. 1B) indicated otherwise, highlighting distinct host factor protein translation mechanisms in the different cell types. Finally, immunofluorescence analyses of ACE2 and BSG/CD147 confirmed the western blot data showing low to no ACE2 expression in iPS cells and mesoderm and increased expression levels in IM cells and podocytes (Fig. 1E and Extended Fig. 1). An inverse profile was observed for BSG/CD147, with the highest expression level in iPS cells and the lowest in podocytes (Fig. 1E).

**Fig. 2:**
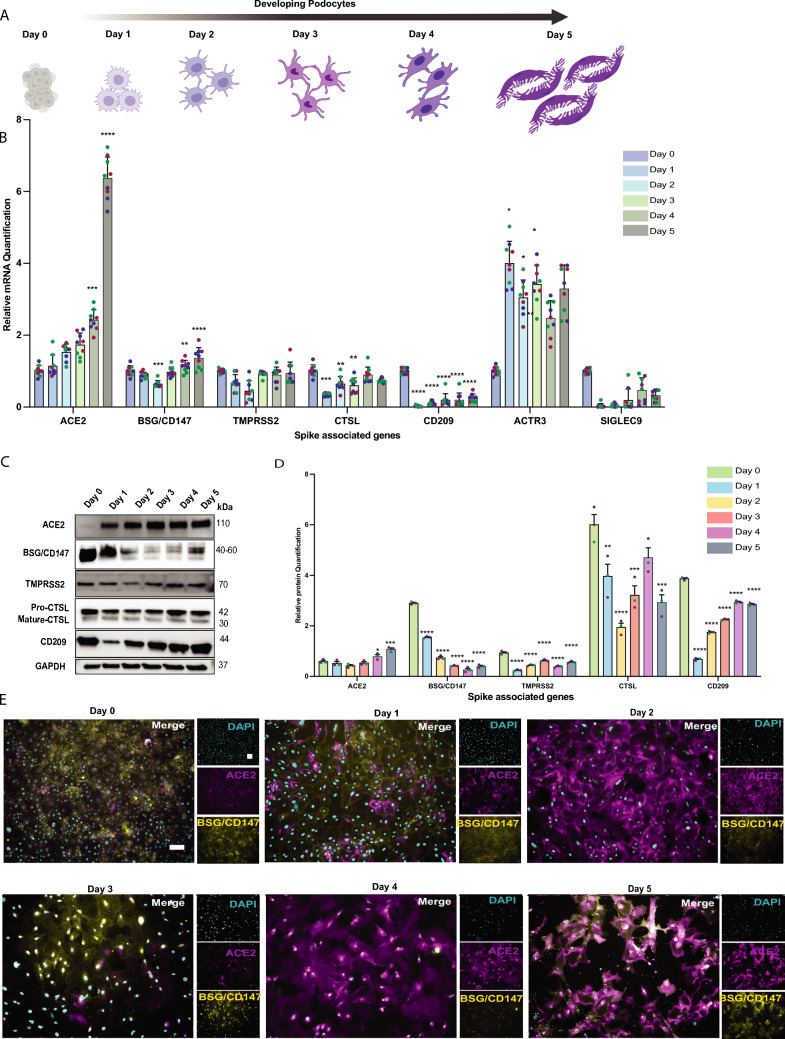
Analyses of spike-interacting factors in developing and mature podocytes. **A** Schematic overview of IM cells and podocytes at different developmental stages (developing podocytes). **B** qPCR quantification of ACE2, BSG/CD147, TMPRSS2, CTSL, ACTR3, CD209, and SIGLEC9 in the cell types generated from the developing podocytes (normalized to IM cell group, denoted Day 0). One-way analysis of variance (ANOVA) with Sidak’s multiple comparison test was used to determine statistical significance. Only p-values of 0.05 or lower were considered statistically significant (p > 0.05 [ns, not significant], p < 0.05 [*], p < 0.01 [**], p < 0.001 [***], p < 0.0001 [****]). Error bars indicate ± SEM (standard error of the mean). Representative **C** western blot with **D** densitometric quantification of three biological replicates to evaluate protein level expression of ACE2, BSG/CD147, CD209, TMPRSS2 and CTSL in the different cell types generated from developing podocyte; GAPDH was used as a loading control. **E** Immunostaining analysis of ACE2 (magenta) and BSG/CD147 (yellow) expression in the cell types generated from developing podocytes and counterstained with DAPI (cyan). Scale bar: 100 µm. Figure **A** was created with Biorender^TM^

Given the observation that most of the spike-associated factors diverge between IM cells and podocytes, we performed time-course analysis to evaluate the expression of the genes on each day of podocyte differentiation. From the protocol, differentiation from IM cells to podocytes takes 5 days (Fig. 2A). We hypothesize that with each continued day of subjecting the cells to the differentiation medium, there will be a change in expression of key factors as we also observe morphological changes in the cells as they mature (Supplementary Fig. S1B). Interestingly, we observed a daily increase in the mRNA level expression of ACE2 and CD209, but the increase was observed after Day 1 for CTSL and Day 2 for BSG/CD147 (Fig. 2B). There was no change in the expression of TMPRSS2 mRNA as cells differentiated from the IM stage (day 0) to day 5 podocytes. We then confirmed the gene expression at the protein level using western blot analysis (Fig. 2C), with blot quantification (Fig. 2D) and immunofluorescence staining for ACE2 and BSG/CD147 (Fig. 2E). The protein level expression for ACE2 and CD209 was consistent with the mRNA expression pattern in Fig. 2B. As expected, there was no significant difference in protein level expression for TMPRSS2. However, for BSG/CD147, although there was a daily increase in mRNA levels, the opposite was true for protein levels. This observation is similar to those in Fig. 1B and D. Consistent with these observations and hypothesis, the immunofluorescence data shows an increase in ACE2 expression and a decrease in BSG/CD147 expression in the developing podocytes with each day of differentiation or maturation (Fig. 2E and Extended Fig. 2).

### Host Factor Expression Levels Determine Cell Lineage Susceptibility to Viral Uptake

**Fig. 3:**
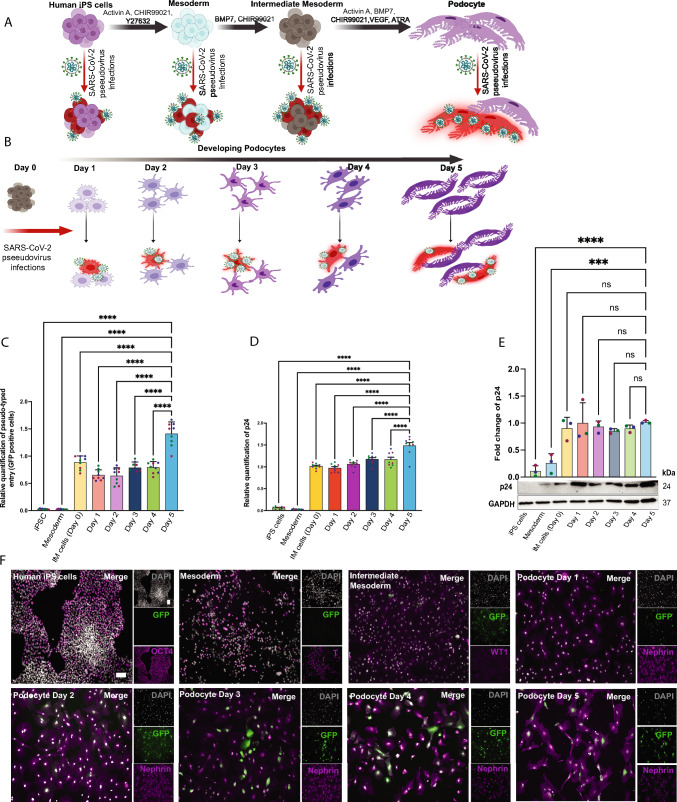
SARS-CoV-2 S-pseudovirus infection of human iPS cell-derived lineages. **A** Schematic overview for S-pseudotyped virus infection of different cell lineages generated using the differentiation protocol illustrated in Fig. 1A. **B** Schematic overview for the S-pseudotyped virus infection of podocytes at different developmental stages. **C** qRT-PCR data quantifying the levels of viral uptake by the cells through GFP using GFP-specific primers in the differentiated podocytes. The data shows an increase in the uptake of the virus from IM cells to podocytes with almost no uptake in iPS cells and mesoderm. **D** qRT-PCR data measuring the p24 levels using GAG-p24-specific primers in the developing podocytes to quantify viral uptake at varying cell maturation states. **E** Representative Western blot with densitometric quantification of three biological replicates from cell lysates of all cell lineages, from iPS cells, mesoderm, IM cells, and immature (Day 0–3) and mature (Day 4–5) podocytes confirming the HIV viral protein p24 visible in permissive cells but not in human iPS cells but minimally in mesoderm cells. GAPDH was used as a loading control. **F** Immunofluorescent images showing GFP (green) and cell lineage identification markers for pseudotyped virus-infected cells. Respective lineage markers are shown in magenta; Oct4 (human iPS cells), Brachyury (Mesoderm), WT1 (IM cells), Nephrin (developing and mature podocytes). Cells were counterstained with DAPI (cyan). Scale bar: 100 µm. The statistical analysis for **C**, **D** and **E** were performed using One-way ANOVA with Sidak’s multiple comparison test. Error bars indicate ± SEM (standard error of the mean). Only p-values of 0.05 or lower were considered statistically significant (p > 0.05 [ns, not significant], p < 0.05 [*], p < 0.01 [**], p < 0.001 [***], p < 0.0001 [****]). Figures **A** and **B** were created with Biorender^TM^

Given the observed differences in the expression of SARS-CoV-2 host factor mRNA and proteins, we wondered if these could translate into differences in viral infectivity or susceptibility in the different cell lineages. To test this, we evaluated SARS-CoV-2 infection using the S-pseudotyped virus exposed to each of the four different cell lineages (Fig. 3A). We also infected IM cells (Day 0) and developing podocytes obtained from each additional day of induction (Days 1–5) with the S-pseudotyped virus (Fig. 3B). To verify viral uptake by the cells, we quantified GFP mRNA transcript levels from undifferentiated and differentiated cells (progenitor and mature podocytes) by qRT-PCR using uninfected cells as controls (Fig. 3C). We observed significantly higher levels of viral uptake as the cells matured. Quantification of the viral particle’s p24 mRNA in the infected cells confirmed the GFP expression results (Fig. 3D). To further verify viral uptake by the cells, we performed western blot analysis and probed for Gag-p24 (present in the pseudovirus’ HIV [32] backbone) taken up by the cells after 48hrs infection (Fig. 3E). From Fig. 3E, the p24 band that corresponds to viral particles taken up by the cell is slightly visible from the mesoderm stage during differentiation although a more pronounced band is visible from the IM stage indicating only a minor uptake in the mesoderm. Densitometric quantification of three independent western blots revealed a significant increase in viral particle uptake from human iPS and mesoderm cells to the mature podocytes (day 5 podocytes) (Fig. 3E). These results confirm that pre-IM progenitor cells are less permissive to SARS-CoV-2 binding and uptake. Although cells with little or no ACE2 expression can still be infected with SARS-CoV-2 [5, 33] through other binding factors, human iPS cells did not take up the viral particles even though they expressed several other host factors [12] (Fig. 1). While human iPS cells express high levels of BSG/CD147 (and almost no ACE2), they were still not permissive to the virus (Fig. 3C, D and E). Additional characterization of the cells via fluorescence microscopy for viral GFP expression (Fig. 3F, see additional images in Extended Fig. 3) indicated no GFP-positive population in human iPS cells and very few GFP-positive cells in the mesoderm lineage. GFP-positive cells were readily detected in IM cells and podocytes at early and late differentiation timepoints, which confirms high susceptibility of these differentiated cell types to the viral particles.

### Viral Uptake Modifies Host Factor Gene Expression in Developing Podocytes

**Fig. 4:**
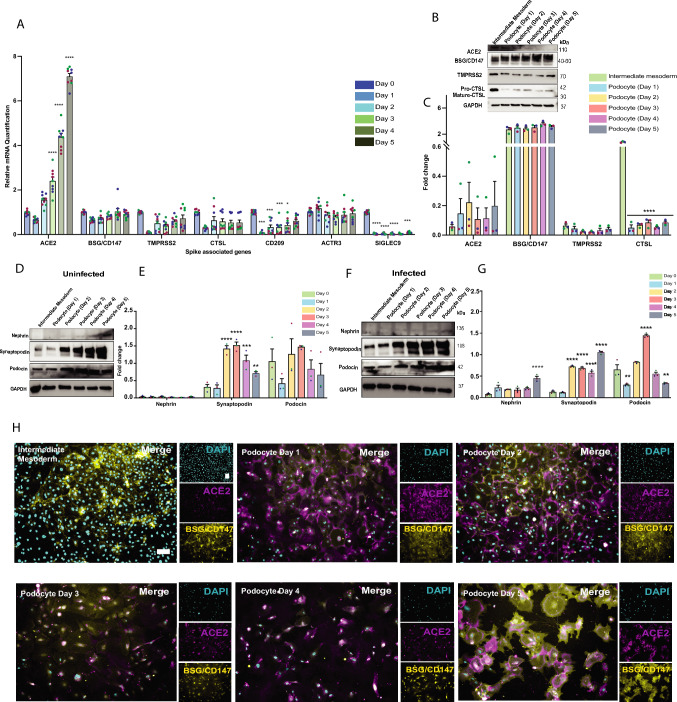
Examining S-pseudotyped virus infection on immature and mature podocytes. **A** qPCR quantification of ACE2, BSG/CD147, TMPRSS2, CTSL, ACTR3, CD209, and SIGLEC9 in infected cell types generated from developing podocytes (normalized to uninfected IM cell group at Day 0 of podocyte induction stage). One-way analysis of variance (ANOVA) with Sidak’s multiple comparison test was used to determine statistical significance. Only p-values of 0.05 or lower were considered statistically significant (p > 0.05 [ns, not significant], p < 0.05 [*], p < 0.01 [**], p < 0.001 [***], p < 0.0001 [****]). Error bars indicate ± SEM (standard error of the mean). Representative **B** western blot with **C** densitometric quantification of three biological replicates to evaluate changes in protein expression of ACE2, BSG/CD147, CD209, TMPRSS2 and CTSL in the developing and mature podocytes after infection with S-pseudotyped virus; GAPDH was used as a loading control (Representative Western blot with densitometric quantification of podocyte-specific proteins in **D** uninfected blot with their **E** quantifications and **F** infected blot with **G** quantification. Samples revealed downregulation of podocin with sustained expression of synaptopodin (SYNPO) and Nephrin after S-pseudotyped virus infection in the cells. **H** Immunostaining analysis of ACE2 (magenta) and BSG/CD147 (yellow) expression in the infected cell types generated from podocyte induction. The cells were counterstained with DAPI (cyan). Scale bar: 100 µm

Next, we examined whether differences exist in SARS-CoV-2 host factor expression as developing and mature podocytes become infected with viral particles. We checked genes (Fig. 4A) and protein (Fig. 4B) level expression in the infected cells. While ACE2 mRNA levels continued to increase with podocyte differentiation and maturation, the protein level analysis shows a decrease with time and cell maturation, although quantification of the data suggested no statistically significant difference. These results are consistent with previous studies showing altered ACE2 expression in diseased or infected murine lung cells [34] and Vero E6 cells [35]. For BSG/CD147, the mRNA and protein levels correlate showing that there is an increase in BSG/CD147 with increasing differentiation and maturation state of the infected samples. This expression trend between ACE2 and BSG/CD147 in podocytes is interesting. It should be noted that in the uninfected samples, we observed that ACE2 protein increases and BSG reduces while in infected samples, ACE2 reduces and BSG increases as the cells become more differentiated. For CTSL, mRNA expression in the developing podocytes correlates with protein expression. Day 0 (IM cells) shows the highest CTSL expression, and a decrease was observed in CTSL expression from Day 0 to 1 and no change between Days 1 and 5 of differentiation (Fig. 4A and B). CD209 mRNA levels also show a decrease from Day 0 to Day 5. While ACTR3 mRNA shows no changes between the developing podocytes, SIGLEC9 mRNA levels show a decrease in the cells. There is no significant change in TMPRSS2 mRNA and protein expression. Together, the observed protein level changes in host factor and processing enzyme expression in infected podocytes may suggest high receptor/enzyme activity and turnover rates as the cells bind and process viral particles.

Podocyte injury and the progression of podocytopathies are often associated with changes in the expression levels of cell lineage-specific biomolecules [36–38]. While the cells looked morphologically similar (Supplementary Fig. S2C), we observed significant changes in podocyte lineage marker expression before and after viral infection. By comparing the mRNA (Supplementary Fig. S2A and B) and protein (Fig. 4D–G) expression levels of podocyte lineage identification markers between uninfected (Fig. 4D and E) and infected (Fig. 4F and G) cells at varying stages of differentiation (Day 0 to Day 5 podocytes), we observed an increase in nephrin and synaptopodin and a decrease in podocin upon infection with the viral particles. These changes in the expression of key podocyte markers could indicate maladaptive response to viral infection or pathogenesis.

We previously revealed that infection with live SARS-CoV-2 leads to dynamic changes in the expression of podocyte lineage identification genes [12]. In this study, we examined changes in the mRNA and protein expression before and after exposure to the S-pseudotyped virus. We analyzed expression of iPS cell markers (Oct4 and Nanog) between uninfected and infected iPS cells and observed no significant difference in the pluripotency marker Oct4 but a statistically significant difference in Nanog expression (Supplementary Fig. S2D). Phase contrast images also did not show a significant morphological difference between infected and uninfected human iPS cells (Supplementary Fig. S2E).

Because viral infections like SARS-CoV-2 can induce pathological effects on cells and tissues even in the absence of direct infection [39], it is possible that progenitor cells that are not directly infected by the virus may still be damaged or diseased through secondary responses including cytokine storm or inflammatory cell death. While there was a slight significant change in Nanog (one of the iPS cell lineage markers) upon infection with the virus (Supplementary Fig. S2D), mesoderm cells infected with the virus exhibited a significant decrease in their lineage identification markers (Supplementary Fig. S2F). Additionally, we observed a significant reduction in cell division/cell growth as seen by the number of mesoderm cells in the plate after 48 h of viral infection (Supplementary Fig. S2G). Immunofluorescence analysis confirmed the above observations in the mature podocytes (Fig. 4H and Extended Fig. 4).

### Microfluidic Organ Chip Devices Model SARS-CoV-2 Glomerular Infections Mediated by the Vasculature

**Fig. 5:**
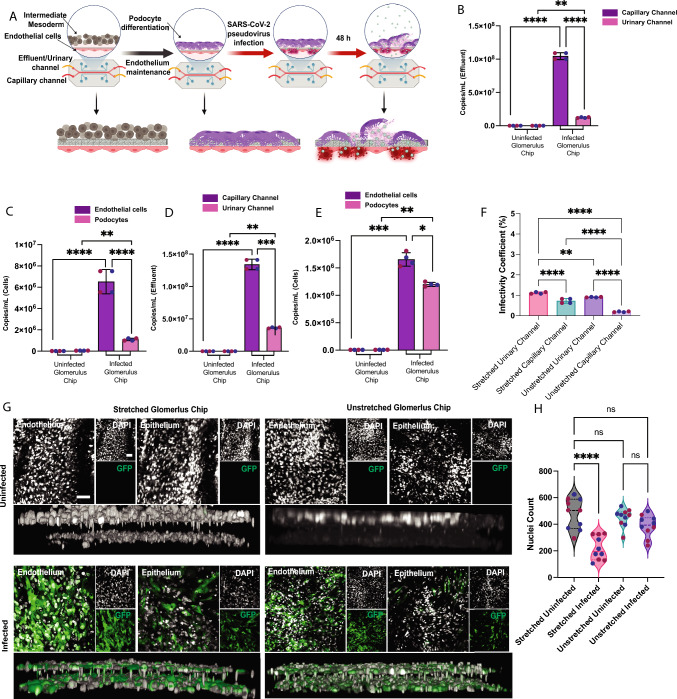
SARS-CoV-2 S-pseudovirus infection of organ chip model of the human kidney glomerulus. **A** Schematic overview glomerulus chip infection with S-pseudotyped virus. qRT-PCR analysis using Lenti-X titration kit for the **B** effluent and **C** cells for mechanically stretched microfluidic glomerulus chip, and **D** effluent and **E** cells for unstretched glomerulus chip. The data revealed that the S-pseudotyped virus crossed the filtration barrier at the vascular-epithelial interface to infect the podocyte layer after vascular delivery of viral particles. **F** Infectivity coefficient (%) comparing viral uptake for capillary/vascular and urinary/podocyte channels in both stretched and unstretched channels revealing a significant increase in uptake of viral particles in the urinary channel (podocytes) compared to capillary channel (vascular endothelium) for both stretched and unstretched glomerulus chips. **G** Fluorescent images of the glomerulus chips showing DAPI, GFP and merged displays for pseudotyped infected chips and uninfected controls for the mechanically stretched and unstretched conditions. **H** Nuclei count of representative fields of view between infected and uninfected urinary channel in the stretched versus unstretched glomerulus chips. Scale bar: 100 µm. One-way analysis of variance (ANOVA) with Sidak’s multiple comparison test was used to determine statistical significance. Only p values of 0.05 or lower were considered statistically significant (p > 0.05 [ns, not significant], p < 0.05 [*], p < 0.01 [**], p < 0.001 [***], p < 0.0001 [****]). Error bars indicate ± SEM (standard error of the mean). Figure **A** was created with Biorender^TM^

Up to this point, our data with 2D cultures provided information about the expression of markers and the uptake of the virus, but 2D culture does not fully mimic in vivo processes. In vivo, viral infections in the glomerular epithelium occur via blood flow through the glomerular capillaries. The processes cannot be fully recapitulated in standard tissue culture systems because they lack the fluid circuit and endothelium-lined vasculature present in the intact glomerulus. Microphysiological systems such as the kidney glomerulus chip provide powerful avenues to address these limitations given their ability to incorporate multiple cell types (such as the podocytes and endothelial cells) in the appropriate structure or tissue pattern and simultaneously provide perfusable microenvironments to mimic the flow of blood and other biological fluids. Moreover, relative to 2D culture, 3D glomerulus chip systems show advanced levels of structural and functional maturation, including specialized foot processes that form interdigitations and in vivo-like deposition of basement membrane proteins including collagen type IV [23]. To develop a more physiologically relevant system that better recapitulates tissue-tissue interfaces of the glomerular capillary wall, we engineered a microfluidic organ chip device that comprises human stem cell-derived isogenic epithelial (podocyte) and endothelial cells. To model SARS-CoV-2 organ tropism, which broadly occurs through circulating body fluids, we infected the glomerular chip with the S-pseudotyped virus through the vascular compartment of the chip lined by the isogenic endothelium interfaced with the glomerular epithelium (podocyte layer) across a flexible porous membrane. We examined whether the S-pseudotyped virus could infect the podocyte layer in the 3D chip model of the kidney glomerulus (Fig. 5A). To accomplish this, we first seeded human iPS cell-derived intermediate mesoderm cells in the top channel of the chip and then differentiated them in situ with fluid flow using podocyte differentiation media. Because mechanical strain can mimic the stretch and relaxation motions of the glomerular capillaries and enhance podocyte development and functional maturation [23], we evaluated the effect of mechanical strain on SARS-CoV-2 infection in the glomerulus chip.

We inoculated the glomerulus chips with the S-pseudotyped virus through the vascular channel. After 48 h of exposure to viral particles, we collected the effluent and harvested the different cell types (endothelium and epithelium) to examine their susceptibility to the virus. The total viral RNA in the effluent and cells from each fluidic channel of the microfluidic chip were quantified using the Lenti-X qRT-PCR titration kit. The copies mL^−1^ of viral RNA from the effluent (Fig. 5B) of the stretched glomerulus chip in both channels indicate movement of the viral particle to the urinary channel. Additionally, the podocytes also show significantly increased viral copies mL^−1^ compared to the uninfected control (Fig. 5C) indicating that podocytes do take up the virus even though the infectious particles were introduced in the vascular compartment. This establishes the infectivity of the chip and demonstrates that the glomerulus chip can be used to model the dynamics of SARS-CoV-2 infection in vivo through the vasculature. Additionally, we collected samples from the glomerulus chip that were not subjected to mechanical strain (unstretched), and we observed the presence of the viral particles in the effluent in both capillary and urinary channels (Fig. 5D) also showing the movement of the viral particles through the porous membrane separating the two cell types or adjacent cell layers. The podocyte layers in the unstretched glomerulus chips also showed a significant increase in viral RNA copies mL^−1^ compared to uninfected glomerulus chips (Fig. 5E). This suggests that pulsatile fluid flow from the stretching motion is not the primary means by which the podocytes get infected. It is important to note that the observed S-pseudotyped infection in podocytes (epithelium) in the stretched and no stretch conditions suggests that viral particle infection in the engineered glomerular tissue results from vascular access and tissue-tissue (endothelial-podocyte) interactions. We then compared 2D (plate culture of Day 5 podocytes) with 3D cultures (stretched and unstretched) and observed that the 2D culture has significantly more viral RNA copies (before normalization) than the 3D culture conditions (Supplementary Fig. S3A). This result was expected because, unlike in vivo, the 2D cultured cells were directly and constantly exposed to viral particles (no vascular perfusion) for 48 h, allowing for constant contact with the uptake receptors. In contrast, in the glomerulus chip (similar to in vivo dynamics), infectious viral particles typically reach cells and tissues via the vasculature and remain in circulation or continuous flow, which also reduces the residence time of viral particles and potentially the rate of infection for a given cell and tissue type. To determine the viral infectivity coefficient, we calculated the ratio of viral particles taken up by the cells to the amount of viral particles available under each condition (in the epithelial and vascular channels, and in standard tissue culture conditions) (Supplementary Fig. S3B). Comparing the viral uptake between urinary channel (podocyte layer) and capillary channel (endothelium layer) in both stretched and unstretched glomerulus chip revealed a higher infectivity coefficient in the urinary channels (Fig. 5F). This means that although the viral particles were introduced through the capillary channel (where the endothelial cells were exposed to more viral particles than the podocytes), the podocytes in the urinary channel exhibited high infectivity under the normalized conditions. This observation is interesting because it reveals an increased susceptibility to viral infection in the podocytes of the glomerulus chip compared to the vascular endothelium. The infectivity coefficient of 2D plate cultured podocytes (direct exposure without vascular access) was predictably high due to the static and direct incubation of the cells without fluid flow (Supplementary Fig. S3C). Taken together, the results from the study indicate that podocytes take up the virus whether exposed directly (as in tissue culture plates) or through the vascular compartment of a more complex and dynamic in vitro model (organ chip).

Immunofluorescent imaging of the glomerulus chips showed a considerable number of GFP-positive cells in the infected chips (for both stretched and unstretched conditions), demonstrating infection in both the vascular and epithelial compartments of the chips (Fig. 5G). Podocyte structure is linked to their function, and as such, defects in their structure as a result of pathogenic infections or disease can cause cell detachment from the glomerular basement membrane and subsequent loss of proteins into the urine, leading to glomerulopathies [40, 41]. We observed considerable loss of podocytes in the glomerulus chip upon viral infection. Calculated nuclei count per ten fields of view revealed significantly fewer cells in the infected glomerulus chips that were more mature or exposed to mechanical strain compared to unstretched/immature chips (Fig. 5H). This result correlates with the infectivity coefficient that shows that the stretched glomerulus chips were significantly more infected than the unstretched (Fig. 5F). This result provides additional confirmation that mechanical strain increases podocyte maturity and the more mature the cells are, the more infected they become upon exposure to viral particles. It has been previously shown that SARS-CoV-2 infection activates necroptosis and apoptosis pathways in podocytes [12, 42]. Thus, cell loss in the infected samples is a direct consequence of virus-induced cytotoxicity.

## Discussion

Human iPS cells and their differentiated derivatives can advance translational research and understanding of patient-specific responses to diseases. Direct involvement of the kidneys in COVID-19 disease has been indicated by multiple reports showing the presence of SARS-CoV-2 RNA in infected tissues from patients [43], and several studies indicate that stem cell models of the kidney can be infected by the virus. Nonetheless, there exists a gap in understanding the susceptibility of progenitor cell types, where some only express a receptor or protease enzyme. It should be noted that when studying diseases and their mechanisms, it is important to employ developmentally and functionally appropriate models. In the case of viral or pathogenic SARS-CoV-2 infections, it is also critical to use disease models that express both entry receptors and processing enzymes. In this study, we generated kidney progenitors as well as mature podocytes [23] such that the progenitor cells represented early stages of nephrogenesis whereas the more specialized podocytes/epithelium modeled features of the mature human tissue. Several groups have utilized kidney organoid models to study SARS-CoV-2 pathogenesis [20, 21]. However, existing kidney organoid models comprise immature cell types that do not recapitulate the tissue-tissue interface and function observed in vivo. To address these limitations, we designed a vascularized organ chip platform by interfacing key cell types of the kidney glomerulus—podocytes and vascular endothelial cells—to recapitulate the tissue structure of the glomerular capillary wall.

Studies of SARS-CoV-2 infection and pathogenesis using primary cells is challenging due to limited tissue supply, and immortalized cells have dedifferentiated phenotypes which diminish their biological relevance and capacity to model in vivo-like processes. In this study we established tissue-specific viral tropism in kidney glomerular models at the cellular and tissue levels. We confirmed the expression of key coronavirus associated genes in the different cell lineages, including mature and immature podocytes. We built on our prior findings that ACE2, BSG/CD147, Cathepsin L (CTSL), CD209 and very low levels of TMPRSS2 are detected in podocytes and showed here that expression levels of these genes differ considerably in undifferentiated and progenitor or less specialized cell lineages. We have initially demonstrated that SARS-CoV-2 can productively infect human iPS cell-derived podocytes by utilizing ACE2 and BSG/CD147. While the reason for the inverse relationship between gene and protein level expression of BSG/CD147 in podocytes is yet to be fully explored, the relatively lower protein expression levels could arise from rapid turnover due to prominent BSG/CD147 receptor involvement in viral binding and internalization in podocytes which may not necessarily be required or utilized in the other cell types. We evaluated the permissiveness of human iPS cells and kidney progenitor cells to SARS-CoV-2 infection by infecting them with S-pseudotyped virus (Fig. 3A and B). Since ACE2 expression is limited to the main site of infection of SARS-CoV-2, it is not surprising that uptake of the virus was absent in human iPS cells as these cells lack ACE2 (Fig. 3C and D) suggesting that the lack of ACE2 expression is one of the determinants of SARS-CoV-2 non-vulnerability in these cells. Although BSG/CD147 serves as a host factor for viral uptake in podocytes [12], its high protein expression in iPS cells does not lead to an uptake of the virus as no GFP-positive cells were detected after inoculation with the viral particles. These results indicate that BSG/CD147 does not facilitate viral uptake in the undifferentiated human iPS cells.

A previous study showed peak levels of ACE2 expression during erythropoiesis, which makes erythroid progenitors more vulnerable to infections by SARS-CoV-2 [6]. IM cells (kidney progenitor cells) have a high expression of ACE2 and, like early erythroid cells, might also serve as target cells for infection in developing kidney tissues. It is established that ACE2 receptor and TMPRSS2 enzyme are important entry receptor and priming protease for SARS-CoV-2 in host target cells [44], and additional receptors such as BSG/CD147, NPR1, CD209 have also been shown to facilitate entry of the virus into target cells [9, 12, 17, 18]. However, concurrent expression of both ACE2 and TMPRSS2 is required to drive viral uptake by the cell [45]. There is no doubt that in human iPS cells, SARS-CoV-2 cell entry is dependent on the concurrent expression of ACE2 and TMPRSS2. Thus, ACE2 expression is likely required to activate SARS-CoV-2 cell entry and processing machinery in these cells. However, podocytes express higher levels of ACE2 when compared to TMPRRS2 expression and we have confirmed previously that BSG/CD147 is also used by the virus to gain access into podocytes. Additionally, our data indicates that the permissiveness of the examined cells to the virus depends on the cell’s level of maturity and specialization. Therefore, existing human iPS cell-derived kidney organoids possessing immature glomeruli [46], are not ideal to recapitulate the biology of SARS-CoV-2 infection in adults. These findings also indicate that IM cells were permissive to SARS-CoV-2 and might support productive virus replication. In line with these data, we propose that immature or progenitor cells will be at a moderate risk for infection and mature cells more accurately predict the devastating effects of SARS-CoV-2 on human kidney health. These indications are also consistent with the concept that early embryonic and placental development are at moderate risk of infection when compared to specialized adult tissues [5].

We employed a mature isogenic glomerular capillary wall by harnessing the power of stem cell biology and microfluidic organ-on-a-chip technology. Using a microphysiological environment that recapitulates features of the adult glomerulus, we investigated whether the podocytes were permissive to SARS-CoV-2 infection. We challenged the capillary channel with the S-pseudotyped virus and detected the viral RNA in the urinary channel (Fig. 5B and D), thereby indicating that the virus must have traversed the permeable basement membrane components separating the vascular and epithelial tissues to reach and infect the podocytes (Fig. 5C and E), as expected in vivo. The glomerulus chip provides a more physiological 3D environment in which the virus moves through the endothelial layer to the urinary channel to infect the podocytes. Applying mechanical strain to the glomerulus chip (which is known to make the podocytes more mature) confirmed that the more mature the cells are, the more susceptible they become to the virus (Fig. 5F). Our results underscore the importance of selecting developmentally accurate models when trying to study the cellular and tissue-level effects and mechanisms of SARS-CoV-2 infection, as the expression of key uptake receptors can be absent or differentially regulated in less specialized tissues, causing poor understanding or interpretation of disease pathogenesis in postnatal and adult tissues. Since in vitro systems are traditionally very limited in their capacity to recapitulate adult physiology, the use of microphysiological environments, such as an organ chip system are advantageous for preclinical testing and translational science. Future studies could examine blood filtration function of the specialized glomerulus chip models to help uncover the dynamics of acute and chronic kidney complications observed in patients with severe COVID-19 disease and other viral mediated glomerulopathies, including HIV-associated nephropathy (HIVAN). Together, the experimental strategies and outcomes of this study have implications for dissecting the mechanisms of disease and the development of therapeutic strategies including antiviral drug candidates.

## Conclusion

In sum, this study highlights the critical influence of cellular developmental status and maturation state on the expression of host factors, ultimately shaping cell susceptibility to viral infection. We demonstrate that mature kidney cells exhibit distinct viral uptake dynamics compared to their immature counterparts, underscoring the necessity of using developmentally appropriate models for studying infectious diseases and related health complications. This finding is particularly significant for investigations into adult-onset diseases, where tissues are highly specialized and exhibit unique functional characteristics that are absent in immature or fetal-like models. Failure to account for these developmental differences can obscure key mechanistic insights, leading to misinterpretation of in vitro, in vivo, and clinical data. Researchers relying on immature cellular models may inadvertently overlook crucial aspects of disease pathology, which could contribute to discrepancies between experimental findings and clinical observations. Our work emphasizes that experimental model selection is not merely a technical consideration but a fundamental factor in ensuring the translational relevance of biomedical research.

Moreover, we demonstrate that vascularized microfluidic systems provide a significant advantage by enabling the formation of functionally mature kidney tissue with vascular access, closely mimicking the in vivo environment. This platform facilitates the in vivo-like viral inoculation, enhancing the physiological relevance of infection models and offering a superior in vitro approach for future studies of host-pathogen interactions. Importantly, our findings reveal that mature kidney cells are significantly more susceptible to SARS-CoV-2 infection than their fetal-like counterparts—an observation that would be overlooked in conventional in vitro dish cultures or organoid models that do not fully recapitulate postnatal and adult tissue architecture and function.

Together, these insights reinforce the necessity of refining model systems to align with human physiology more accurately. By leveraging advanced vascularized platforms and prioritizing developmentally appropriate models, researchers can achieve a deeper mechanistic understanding of infection dynamics, ultimately facilitating the development of more effective therapeutic and preventive strategies. Future studies could involve systematic and multiorgan assessment of live SARS-CoV-2 to gain more comprehensive physiological and genetic insights, thereby enhancing the relevance of the findings to human real-life viral infection and disease pathogenesis.

## Materials and Methods

### Human iPS Cell Culture

The PGP-1 human iPS cell line was gifted to S.M by the Personal Genome Project [47] via George Church’s lab at Harvard Medical School exclusively for research purposes under approved material transfer agreements and their use was approved by the institutional review board and the stem cell research oversight committee. Prior to experimentations, the cell lines were tested and found to be free of mycoplasma contamination (Mycoplasma PCR Detection Kit, abm). The cells were also confirmed to be karyotypically normal. Human iPS cells were maintained under feeder-free conditions in polystyrene tissue culture treated six well plates (Corning) coated with hESC-grade Matrigel (BD Bioscience). The cells were fed daily with pre-warmed mTeSR1 medium (Stem Cell Technologies) and maintained at 37 °C and 5% CO_2_. Cells were inspected daily for signs of spontaneous differentiation and differentiated cells were scraped away using standard methods. When the stem cell colonies reached approximately 70% confluence, the cultures were washed twice with Advanced DMEM/F12 (Gibco) and then passaged to fresh Matrigel-coated plates using Accutase (Gibco) and plated at 1:6 splitting ratio.

### Derivation of Podocytes from Human iPS Cells

Podocytes were generated using our established method [23–25]. Briefly, human iPS cells were cultured to 70% confluence on Matrigel-coated plates and then dissociated with warm enzyme-free dissociation buffer (Gibco). Colonies were scraped using a cell-lifter (Fischer Scientific) and then pelleted by centrifuging the cell suspension twice at 200 g for 5 min each in advanced DMEM/F12 (Gibco). The second centrifugation is a critical step that helps to remove residual Matrigel, which can affect differentiation or cell signaling. To generate mesoderm cells, following centrifugation, human iPS cells were resuspended in mesoderm induction media, consisting of DMEM/ F12 with GlutaMax (Gibco) supplemented with 100 ng mL^−1^ Activin A (Invitrogen), 3 μM CHIR99021 (Stemgent), 10 μM Y27632 (TOCRIS), and 1 × B27 serum-free supplement (Gibco). The cells were plated at a seeding density of 100,000 cells per well of a tissue culture treated 12-well plate coated with Laminin 511-E8 (Takara). Cells were then cultured in mesoderm induction media for 2 days with daily media change. For intermediate mesoderm differentiation, at the end of the initial 2-day mesoderm induction period, cells were transitioned to intermediate mesoderm media (containing DMEM/F12 with GlutaMax supplemented with 100 ng mL^−1^ BMP7 (Invitrogen), 3 μM CHIR99021, and 1 × B27 serum-free supplement) for a minimum of 14 days, with daily media change. After 14 days in intermediate mesoderm induction, the cells can either be cryopreserved or differentiated to podocytes. To initiate podocyte induction, the intermediate mesoderm cells were dissociated using 0.25% trypsin-EDTA (Gibco) and then plated at a seeding density of 100,000 cells per well on a freshly prepared laminin-511-E8 coated 12-well plate. The resultant cultures were fed daily for 1 to 5 days (depending on the desired cell maturation state) with a podocyte induction medium consisting of advanced DMEM/F12 with GlutaMax supplemented with 100 ng mL^−1^ of BMP7, 100 ng mL^−1^ of Activin A, 50 ng mL^−1^ of VEGF (Gibco), 3 μM CHIR99021, 1X B27 serum-free supplement, and 0.1 μM all-trans retinoic acid (Stem Cell Technologies). It is recommended to keep this medium protected from light using aluminum foil, given the photoinstability of retinoic acid. Following the daily podocyte induction period, cells were transitioned to maintenance media (Culture Boost-R, Cell Systems) with or without the virus for 2 days prior to endpoint experimentation.

### Vascular Endothelial Cell (viEC) Differentiation from Human iPS Cells

We differentiated viEC from PGP-1 human iPS cells to ensure that the resulting organ chip was isogenic using an established protocol (Atchison et al.) optimized by Roye et al [48, 49]. Human iPS cells at 85% confluency were harvested with Accutase and reseeded at a cell density of 47,000 cells cm^−2^ on Matrigel-coated six-well plates. After 24 h (Day 1), we replaced the mTeSR1 media with N2B27 media containing neurobasal media (Invitrogen) and DMEM/F12 GlutaMax (Invitrogen) at 1:1 ratio and N2 (100×) (Invitrogen) and B27 (−) Vitamin A (Invitrogen) supplemented with 8 μM CHIR-99021 (Reprocell Inc) and 25 ng mL^−1^ hBMP4 (VWR). Cells were cultured in the N2B27 media for 3 days without media change to induce lateral mesoderm cells. On day 4, N2B27 media was replaced with media containing StemPro-34SFM media (Invitrogen) supplemented with 1× Glutamax (Invitrogen) and 1× Pen-Strep (Gibco) supplemented with 2 μM forskolin (Abcam) and 200 ng mL^−1^ VEGF165 (Invitrogen). This is called the endothelial induction media and cells were fed with this daily from day 4 to day 6 with collection of conditioned media on day 5 to day 7. Sorting of viEC was done on day 7 by harvesting CD144^+^ (VE-Cadherin) and CD31^+^ (PECAM-1) cells, as described below.

### Magnetic-Activated Cell Sorting of viEC

After 7 days of endothelial differentiation, cells were individualized using Accutase (incubation at 37 °C for 5–7 min). Cells were collected and neutralized with StemPro-34 media at 1:1 ratio with accutase and centrifuged at 1000 rpm for 5 min. Cells were resuspended and washed with 10 mL MACS buffer [dPBS (Gibco) with 0.5% BSA (MilliporeSigma) and 2 mM EDTA (Invitrogen)] and centrifuged at 1000 rpm for 5 min. the cells were then resuspended in MACS buffer at a density of 80 μL per 10 million cells followed by 20 μL per 10 million cells conjugation with FcR blocking reagent, CD31 Microbeads and CD144 Microbeads (Miltenyl Biotec Inc). The cell suspension was incubated in the dark on ice for 15 min. After ice-incubation, cells were washed with MACS buffer and sorted using QuadroMACS™ Seperator via the magnetic-mediated approach. After sorting, cells were expanded in conditioned media diluted at 1:1 ratio with StemPro-34 SFM supplemented with 2 μg mL^−1^ heparin (STEMCELL Technologies). Media was replaced every 2 days until the conditioned media was fully used. For continued expansion after the first passage, cells were maintained in viEC maintenance media containing StemPro-34 supplemented with 10% heat inactivated FBS (Invitrogen), 2 μg mL^−1^ heparin, and 50 ng mL^−1^ VEGF165.

### Glomerulus Chip Device Functionalization and Cell Seeding

The organ-chip devices (10231-2; LOT 000568) used were purchased from Emulate Inc. (Boston, MA, USA). The chips were activated by treatment with oxygen plasma (100 W, 0.8 mbar, 30 s) using a plasma etcher (Emitech K-1050X). The activated chips were functionalized by incubation with 50 μg mL^−1^ of laminin-511 solution (BioLamina, LN511-0502) in PBS with calcium and magnesium (for both urinary and microvascular channels and both sides of the porous PDMS membrane) and incubated at 37 °C with 5% CO_2_ overnight. Differentiated viECs (9 × 10^4^) were seeded in the macrovascular channel of the chips and incubated by inversion. After 4 h, the channel was flushed with viEC maintenance media to remove non-adherent cells, and the organ-chips were incubated at 37 °C with 5% CO_2_ overnight. The next day, IM cells (8 × 10^4^) were seeded in the urinary channel. After 4 h incubation, both microfluidic channels were flushed with their respective media (Urinary channel—IM induction medium; microvascular channel—viEC maintenance medium) and the chips were incubated at 37 °C with 5% CO_2_ overnight.

### Propagation and Maintenance of the Glomerulus Chip

The reservoirs for the macrovascular and urinary channels of the chip were filled with viEC maintenance media and podocyte induction media, respectively. The pods were primed for 2 min using Zoe-CM1 Culture Module to ensure fluid flow in the inlet and outlet of the fluid circuit. Both fluidic channels were flushed with their respective media (Urinary channel—podocyte induction medium; microvascular channel—viEC maintenance medium) and then were connected to the Pods while maintaining fluid-fluid contact to avoid bubble formation. The Pods were placed inside the Zoe-CM1 (Emulate Bio.) which is connected to an automated vacuum regulator, Orb-CM1 (Emulate Bio.) that directs fluid flow and controls stretch–relaxation cycles. The chip fluidic channels were continuously perfused with the respective cell culture medium at a volumetric flow rate of 60 µL h^−1^ which correlates to a shear stress of 0.0007 dyn cm^−2^ for the urinary channel and 0.017 dyn cm^−2^ for the microvascular channel, along with cyclic strain (10% membrane stretch at an amplitude of ~ 85 kPa and a frequency of 0.4 Hz defined by the manufacturer) [23, 50]. The stretch and relaxation cycles of the porous PDMS membrane mimic the mechanical strain exerted by renal blood flow and pressure in vivo. After 4 days of podocyte differentiation, the podocyte induction media was replaced with CultureBoost-R (Cell Systems) in preparation for viral infection studies described below. For the experiments with unstretched chip conditions, the podocytes and the endothelial cells were perfused with their respective cell-culture media using an Ismatec IPC-N digital peristaltic pump (Cole-Parmer) at a volumetric flow rate of 246 μL h^−1^ (shear stress of 4.09e−3 dyn cm^−2^ for the top channel and 0.07 dyn cm^−2^ for the bottom channel). Cell culture media was recirculated, and fresh media was added to the falcon tubes (reservoirs) every other day. After 4 days, the podocyte induction media was replaced with CultureBoost-R (Cell Systems) before viral infection studies.

### Production of SARS-CoV-2 S-Pseudotyped Lentiviral Particle

For packaging of the S-pseudotyped lentiviral particles, we used the HEK293T cell line (ATCC), which was maintained in HG-DMEM (Thermo Fisher Scientific Inc) supplemented with 10% FBS (Corning). Lentiviral particles were prepared by transiently transfecting HEK293T cells (80–90% confluent in T-75 flasks) with psPax2 (packaging; Addgene plasmid # 12260), pCMV-SCoV-2S (Spike envelope plasmid; Sinobiologicals, VG40589-UT) and pLJM1-EGFP (reporter; Addgene, 19319) at a ratio of 1:1:2, respectively, using the Lipofectamine 3000 kit (Thermo Fisher Scientfic Inc.) according to manufacturer instructions for T-75 flasks, and without antibiotics. After 5 min incubation, the plasmid DNA mix in Opti-MEM- P3000 media was then mixed with the transfection reagent (Opti-MEM and Lipofectamine 3000 reagent) and incubated at room temperature for 10–15 min. Appropriate volumes of transfection mixture were used to transfect HEK 293Tcells in each flask and incubated at 37 °C with 5% CO_2_ for 6 h. After 6 h, the media was changed. Packaged lentiviral supernatants were collected at 24- and 48-h post-media change, after which the supernatants were filtered through 0.45-micron filters (Corning), and ultracentrifuged on a 28% sucrose cushion (Sucrose/PBS; MilliporeSigma) for 3 h at 100,000×*g* at 4 °C. After ultracentrifugation, the supernatant was decanted and resuspended in Tris buffered saline (TBS; Bio-Rad), aliquoted (to avoid freeze/thaw cycles) and then stored at − 80 °C.

### Viral Infection Studies

Infection of the 2D cells were done in the presence of 5 µg mL^−1^ polybrene (MilliporeSigma) for 48 h. For the glomerulus-on-chip, after 4 days of podocyte differentiation, the chips were infected with the S-pseudovirus through the microvascular channel for 48 h. Notably, both 2D and 3D cultured cells were exposed to the same viral MOI. In the microfluidic setup, we accounted approximately 10% loss of signal likely due to residual medium in the microfluidic system tubing connecting the tissue chip to the inlet and outlet reservoirs and through the peristaltic pump. The infectivity coefficients were calculated using the following equations:

1 $${\text{T}}_{{{\text{E}} }} = \left[ {{\text{V}} - {\text{E}}_{{\text{C}}} \left] { \, + \, } \right[{\text{E}}_{{\text{I}}} } \right]$$

2 $${\text{T}}_{{{\text{P}} }} = \left[ {{\text{V}} - {\text{E}}_{{\text{p}}} \left] { \, + \, } \right[{\text{P}}_{{\text{I}}} } \right]$$

3 $${\text{Infectivity coefficient of 3D podocyte }}\left( \% \right) = \left( {\left[ {{\text{P}}_{{\text{I}}} } \right]/{\text{T}}_{{\text{P}}} } \right) \times {1}00$$

4 $${\text{Infectivity coefficient of 3D endothelial cells }}\left( \% \right) = \left( {\left[ {{\text{E}}_{{\text{I}}} } \right]/{\text{T}}_{{\text{E}}} } \right) \times {1}00$$

5 $${\text{Infectivity coefficient of 2D podocytes }}\left( \% \right) = \left( {\left[ {{\text{P}}_{{{\text{TCP}}}} } \right]/{\text{ T}}_{{{\text{TCP}}}} } \right) \times {1}00$$

We calculated the total RNA from virus introduced through the bottom channel [V], endothelial cell uptake [Ei], bottom channel effluent [V − Ec], podocyte uptake [Pi], and urinary channel effluent [V − Ep]. With these values, we further calculated the total endothelial channel infection level as Te = [V − Ec] + [Ei] and the total urinary channel infection as Tp = [V − Ep] + [Pi]. Next we calculated the infectivity coefficients for podocyte and endothelial cells using the following equations:

$${\text{Infectivity coefficient for podocytes }} = \, \left( {{\text{Pi}}/{\text{Tp}}} \right) \times {1}00$$

$${\text{Infectivity coefficient for endothelium }} = \, \left( {{\text{Ei}}/{\text{Te}}} \right) \times {1}00$$

Signal or viral loss was determined by subtracting [Tp + Te] from total viral load [V] administered to the microfluidic organ chip setup or the 2D tissue culture plates. Below is an overview of the terms and variables in the equations used.

[V] = total viral particles introduced into the microfluidic chip, [V − E_C_] = viral particles detected in the outflow of capillary channel, [E_I_] = viral particles detected in the infected endothelial cells, T_E_ = Total signal from the infected capillary channel, [V − E_p_] = viral particles detected in the outflow of the infected urinary channel, [P_I_] = Signal detected in the infected podocytes (epithelial tissue), T_P_ = Total signal from the infected urinary channel, T_TCP_ = Total viral particle introduced into tissue culture plate (2D podocyte model), P_TCP_ = Total signal from the infected podocytes in tissue culture plate (2D podocyte model).

### Gene Expression Analysis

Total RNA was extracted from the cells using the NucleoSpin kit (Macherey-Nagel) according to the manufacturer’s protocol. 5 ng of RNA was used for qRT-PCR using the Luna Universal One-Step RT-qPCR kit (New England Biolabs). Gene expression quantification was performed using the QuantStudio3 96-well, 0.2 mL block instrument (Applied Biosystems) set to detect SYBR with the following thermal cycling steps: 55 °C for 10 min, 95 °C for 1 min and 40 cycles of 95 °C for 10 s and 60 °C for 1 min according to manufacturer’s protocol. Gene expresion fold change was calculated using the double delta CT method, in which samples were first internally normalized to GAPDH housekeeping gene and then normalized to the respective controls (or untreated conditions). Data was compiled and analyzed digitally via the Thermo Fisher Scientific iCloud Relative Quantification Suite. The sequences of the qPCR primers used in this study are provided in Supplementary Table S1. All assays were performed in technical triplicates and at least three independent experiments. Viral RNA from pseudovirus-infected organ chips were also quantified by qRT-PCR using the Lenti-X qRT-PCR titration kit (Clontech) following manufacturer’s instruction.

### Western Blot Analysis

To collect whole cell lysates, cells were washed twice with ice-cold PBS and lysed on ice in RIPA buffer (MilliporeSigma) supplemented with PhosSTOP phosphatase inhibitors (MilliporeSigma) and complete EDTA-free protease inhibitor cocktail (MilliporeSigma). Pierce BCA protein assay Kit (Thermo Fisher Scientific Inc) was used for protein quantification. 15 μg protein lysate was separated by sodium dodecyl sulfate polyacrylamide gel electrophoresis (SDS-PAGE) using Mini-PROTEAN TGX Stain-Free Precast 4–15% stain free gel (Bio-Rad) and transferred using a Trans-blot Turbo semi-dry transfer system (Bio-Rad) onto a PVDF membrane (Bio-Rad). After transfer, the membranes were blocked with 5% milk in Tris-buffered saline with Tween 20 (TBS-T), and immunoblotting was carried out according to standard procedure. Primary antibodies were diluted in TBS-T supplemented with 5% Blotto/TBS-T and incubated overnight in a 4 °C cold room on a platform angle rocker. Primary antibodies used for Western blot were mouse Anti-TRA-1-85/CD147 (R&D systems; MAB3195, 1 µg mL^−1^), goat Anti-ACE2 (R&D systems; AF933, 1 µg mL^−1^), guineapig Anti-Nephrin (Progen; GP-N2, 1:1000), rabbit Anti-Podocin (Abcam; ab50339, 1 µg mL^−1^), mouse Anti-Synaptopodin (D-9) (Santa Cruz Biotechnology; sc-515842, 1:500), mouse Anti-TMPRSS2 (Santa Cruz Biotechnology; sc-515727, 1:500), mouse Anti-DC-SIGN/CD209 (Santa Cruz Biotechnology; sc-65740, 1:500), mouse Anti-Cathepsin L (Santa Cruz Biotechnology; sc-32320, 1:500), mouse Anti-p24 (Santa Cruz Biotechnology; sc-69728, 1:500), rabbit Anti-GAPDH (MilliporeSigma; ABS16, 1:10000). The blots were then washed three times (5 min each) in TBS-T to remove unbound antibodies. Secondary antibodies were then incubated for 1 h in 5% Blotto/TBS-T on a platform angle rocker at room temperature. Secondary antibodies were HRP-conjugated goat Anti-mouse (Cell Signaling; 7076, 1:5000) HRP conjugated goat Anti-rabbit (Cell Signaling; 7074, 1:10000), HRP conjugated rabbit Anti-goat (R&D systems; HAF017, 1:1000) and HRP conjugated goat Anti-guineapig (American Research Products; 90001, 1:1000). Following incubation with secondary antibody, blots were washed thrice for 5 min in TBS-T. Immunoreactive bands were developed using the Super Signal West Femto kit (Thermo Fisher Scientific Inc). The chemiluminescent signals were acquired using a GelDoc Imager (Bio-Rad). All Blue Prestained Protein Standards (Bio-Rad) were used for band referencing and orienting blots during electrophoresis and membrane transfer.

### Immunofluorescence Analyses

Cell fixation and immunolabeling were performed as previously described [12]. Briefly, all primary antibody incubations were performed overnight at 4 °C in a solution containing 0.125% Tween/PBS. Secondary antibody incubations were carried out for 1 h in the dark, at a dilution of 1:1000 in a solution containing 0.125% Tween/PBS. Secondary antibodies used in this study included donkey anti-rabbit Alexa fluor 594 (Life Technologies; A21207, 1:1000), donkey anti-rabbit Alexa fluor 488 (Life Technologies; A21206, 1:1000), donkey anti-mouse Alexa fluor 488 (Life Technologies; A21202, 1:1000) and goat anti-mouse Alexa fluor 700 (Life Technologies; A21036, 1:1000). Cells were counterstained with 4′,6-diamidino-2-phenylindole (DAPI, Invitrogen, D1306). The primary antibodies used were Anti-Nephrin (Progen; GP-N2, 1:250); Anti-Podocin (Abcam; ab50339, 1:250), Anti-WT1 Antibody, clone 6F-H2 (Millipore; 05-753, 1:250), Anti-GFP (Millipore; SAB4301138, 1:1000), Anti-ACE2 (R&D systems; AF933,1:250), Anti-TRA-1-85/CD147 (R&D systems; MAB3195, 1:250), Anti-Cathepsin L (Santa Cruz Biotechnology, sc-32320), Anti-TMPRSS2 (Santa Cruz Biotechnology, sc-515727), Anti-DC-SIGN/CD209 (Santa Cruz Biotechnology, sc-65740), Anti-Brachyury (Abcam; ab20680, 1:250) and Anti-Oct4 (R&D systems; AF1759, 1:250). Images were acquired using an M7000 epifluorescence microscope (Invitrogen) equipped with 10×/0.30 LWDPH with 7.13 mm WD and 20×/0.45 LWDPH with 6.12 mm WD objectives. Immunostained chips were imaged with a Leica SP8 Upright Confocal Microscope, using a 25/0.95 HCXIRAPO water dipping lens with 2.4 mm DIC objective. Images were processed using Fiji software version 2.1.0/1.53c.

### Statistical Analysis

All data were generated from at least three independent experimental replicates with three or more technical replicates each, except otherwise stated. A one-way ANOVA with post-hoc Sidak’s test compared to control was used to test for statistical significance. For all studies, only p-values of 0.05 or less were considered statistically significant (p > 0.05 [ns, not significant], p < 0.05 [*], p < 0.01 [**], p < 0.001 [***], p < 0.0001 [****]). Statistical testing for all studies was performed using GraphPad Prism 9 software package [version 9.5.1 (733)] for Microsoft Windows operating systems.

## Supplementary Information

Below is the link to the electronic supplementary material.

Supplementary file1 (PDF 183 KB)

Supplementary file2 (PDF 2164 KB)

Supplementary file3 (PDF 7076 KB)

Supplementary file4 (PDF 1765 KB)

Supplementary file5 (PDF 10123 KB)

Supplementary file6 (PDF 25820 KB)

Supplementary file7 (PDF 22075 KB)

Supplementary file8 (PDF 14977 KB)

Supplementary file9 (PDF 20501 KB)

Supplementary file10 (DOCX 17 KB)

## Data Availability

The datasets used and/or analyzed during the current study are available from the corresponding author upon reasonable request.
