# Supplementary material for: A Vascularized Human Organ Chip Reveals SARS-CoV-2 Susceptibility in Developmentally Guided Tissue Maturation
Source: Cell Mol Bioeng. 2025 Jul 22;18(5):453–71. doi: 10.1007/s12195-025-00851-4 (PMC12579647; doi:10.1007/s12195-025-00851-4)
Supplement: Supplementary file 1 — Supplementary file1 (PDF 183 KB) [file 12195_2025_851_MOESM1_ESM.pdf]

Supplementary Table 1- Primer sequences used in the study

	**Forward 5' - 3'**	**Reverse 5' - 3'**
ACE2	GGGATCAGAGATCGGAAGAAGAAA	AGGAGGTCTGAACATCATCAGTG
TMPRSS2	AATCGGTGTGTTCGCCTCTAC	CGTAGTTCTCGTTCCAGTCGT
BSG/CD147	ACGTCCTGGATGATGACGAC	CAGGAAGAGTTCCTCTGGCG
CTSL	GGAGAGCAGTGTGGGAGAAG	TGAAGCTGTGTTTCCCTTCC
CD33	CTGCTCACACAGGAAGCCC	AGGGCCCCTGCCCAC
CD209	TGCTGAGGAGCAGAACTTCC	GTTGGGCTCTCCTCTGTTCC
SIGLEC9	GGAGACGGCACAGTATCCAC	AGGGGGATTGCTGTCAACTG
SIGLEC10	TGATCAGAGACGCGCAGATG	GCTTCTGAGTCAGGGCTGTT
ACTR3	CTTCGGCTTCCCGGCTA	AGTTTTGTATACCCCGTGCC
MYO6	AGGATTGCCCAGAGTGAAGC	TTTGGCCATTTGTTCCGGTG
CLEC10A	GAACGGCAGGCAGTTCATTC	GGCATTGTTGTTGAGAGTAGCC
HIV gag	ATCAAGCAGCCATGCAAATGTT	CTGAAGGGTACTAGTAGTTCC
GFP	AGAAGAACGGCATCAAGGTG	GGGTGTTCTGCTGGTAGTGG
Nephrin	GCTTCTGCTCCTCTCCAATG	CCCTGCCTCTGTCTTCTCTG
Synaptopodin	CATTCTGGAGGAGTCGATGG	ATCCAGCAAGTCTGGATTCG
Podocin	TCCACAGAGAAGCCTTCCAC	GCTTCCCTGAGTTCTGTTGC
WT1	GGGTACGAGAGCGATAACCA	TCTCACCAGTGTGCTTCCTG
Pax2	AGATTCCCAGAGTGGTGTGG	CTCAAAGACCCGATCCAAAG
GAPDH	GGAGCGAGATCCCTCCAAAAT	GGCTGTTGTCATACTTCTCATGG
Nanog	AACAATCAGGCCTGGAACAG	CAGGAGAATTTGGCTGGAAC
OCT_4	TATGGGAGCCCTCACTTCAC	AGAGTGGTGACGGAGACAGG
Brachury	CAACCTGGGTACTCCCAATG	ATGAGGATTTGCAGGTGGAC
Goosecoid	CCATCTTCACTGACGAGCAG	TCTCCTGGAAGAGGTTCTCG
