# Supplementary figures and images for: A Vascularized Human Organ Chip Reveals SARS-CoV-2 Susceptibility in Developmentally Guided Tissue Maturation
Source: Cell Mol Bioeng. 2025 Jul 22;18(5):453–71. doi: 10.1007/s12195-025-00851-4 (PMC12579647; doi:10.1007/s12195-025-00851-4)

A

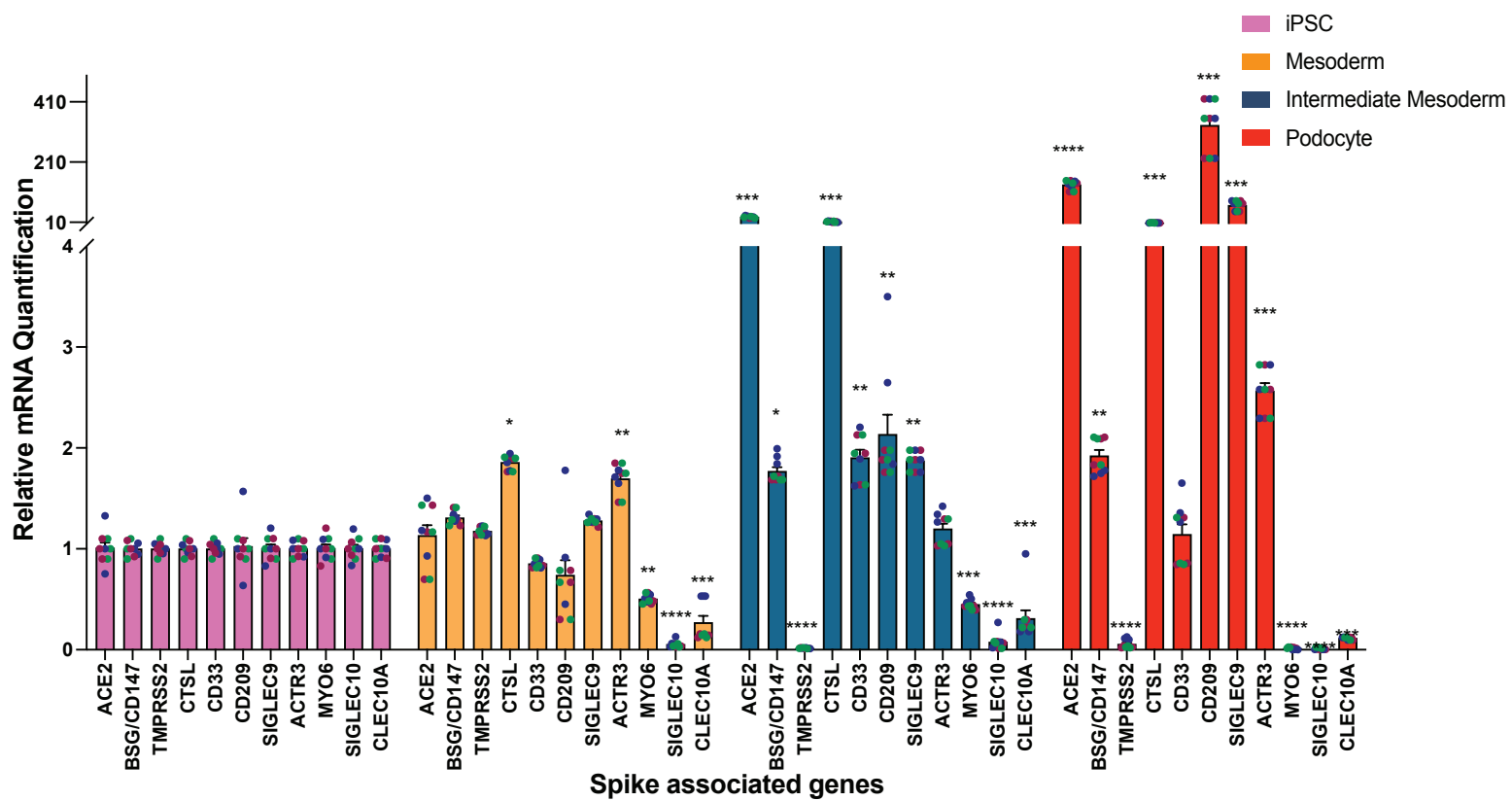

B

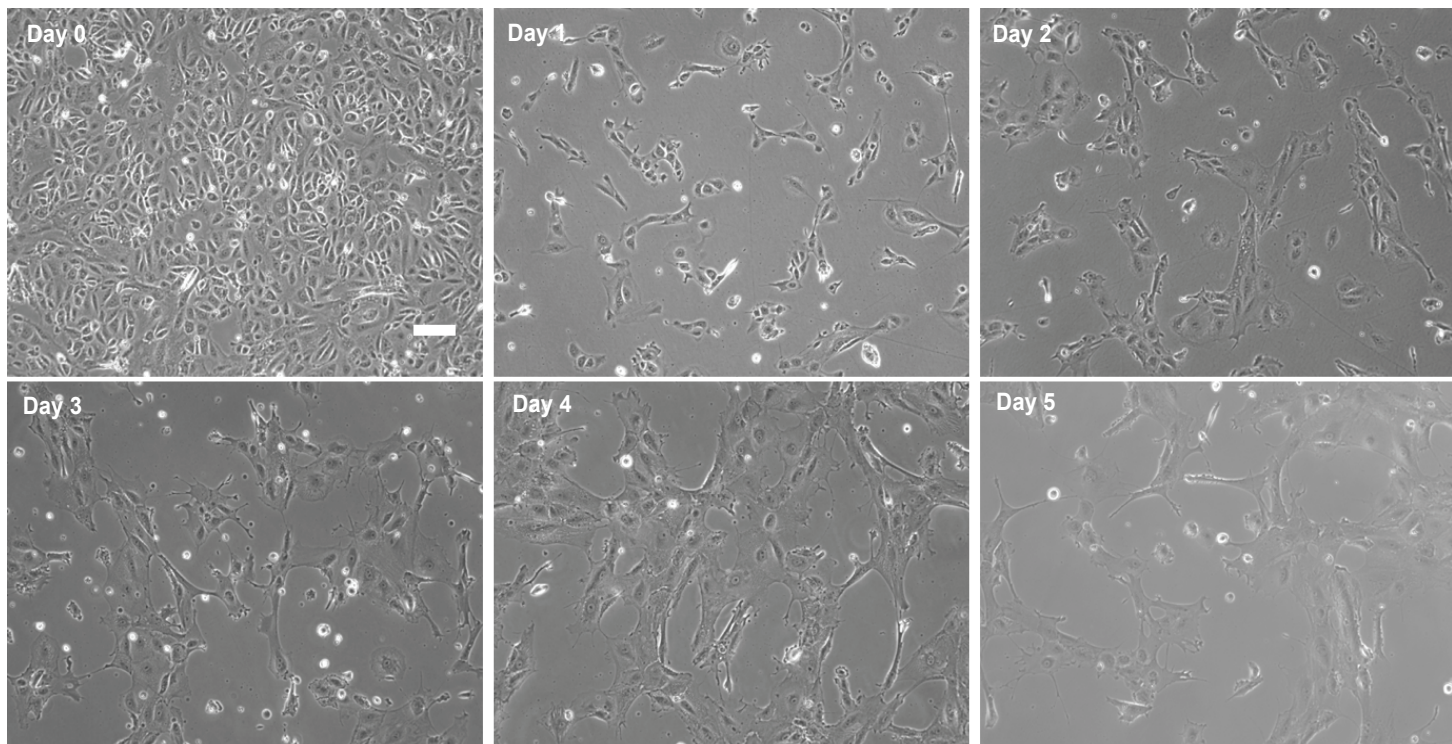

Supplement: Supplementary file 2 — Supplementary file2 (PDF 2164 KB) [file 12195_2025_851_MOESM2_ESM.pdf]

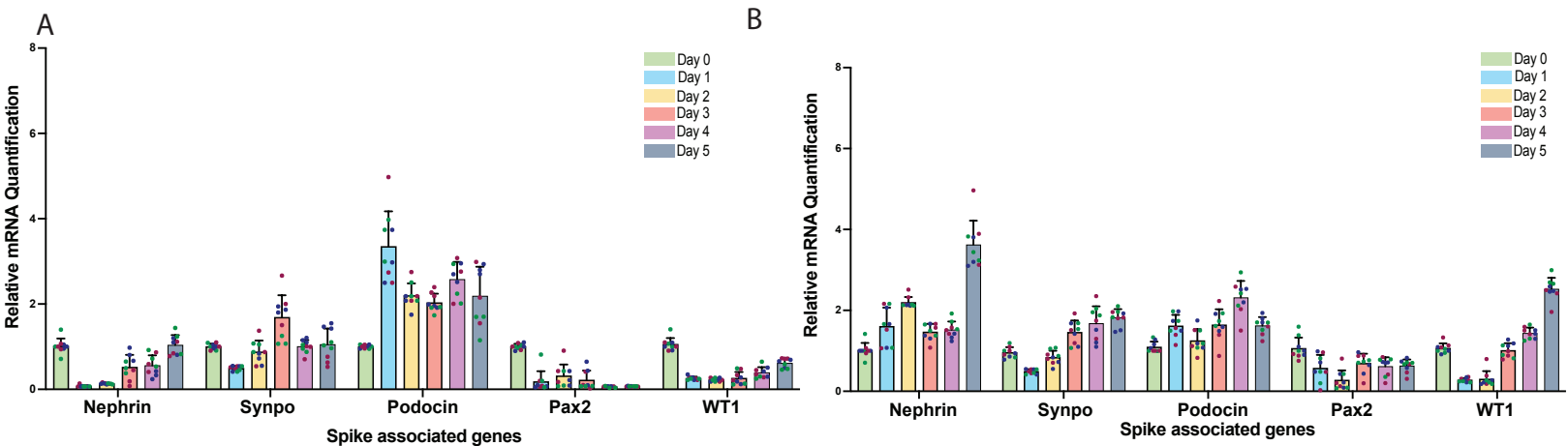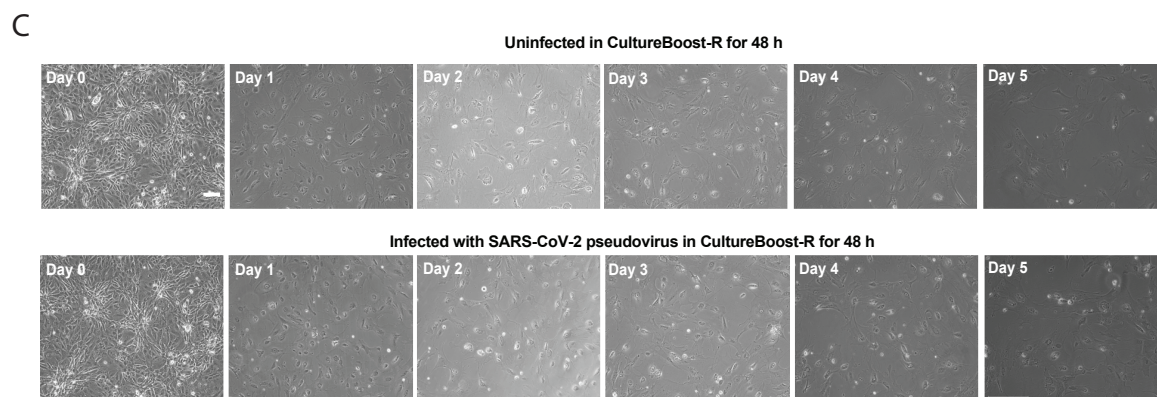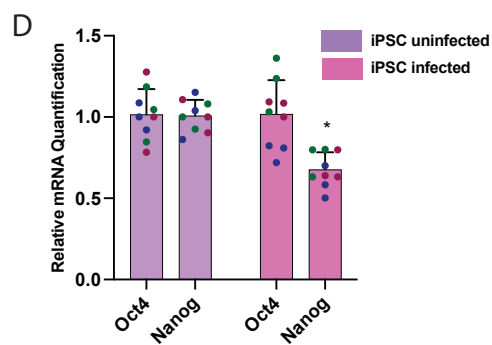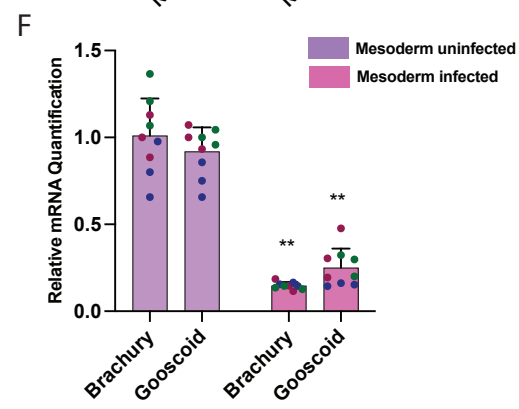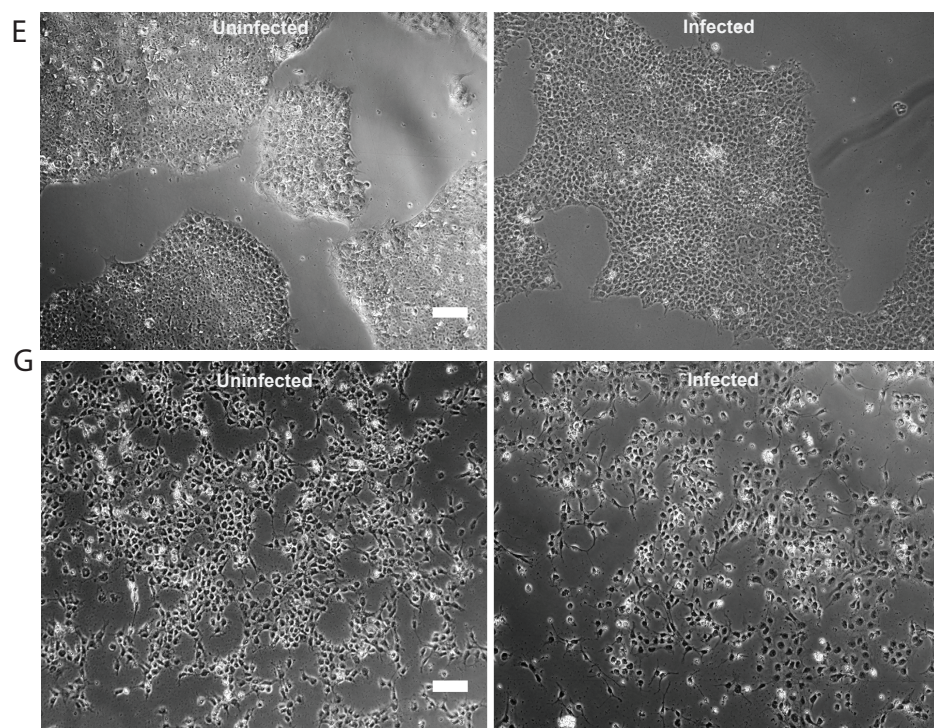

Supplement: Supplementary file 3 — Supplementary file3 (PDF 7076 KB) [file 12195_2025_851_MOESM3_ESM.pdf]

A

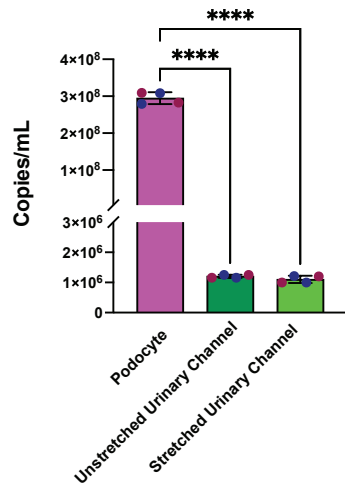

B

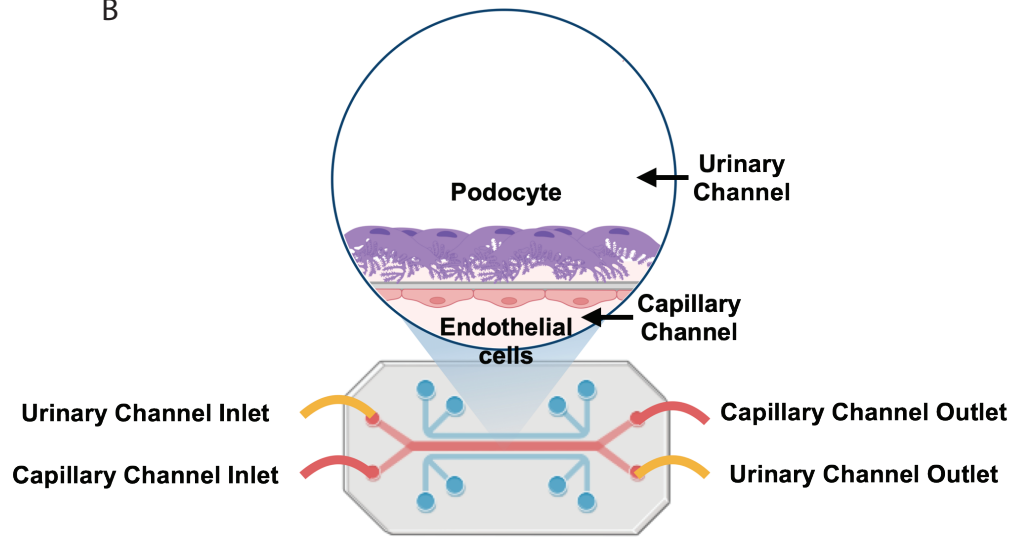

C

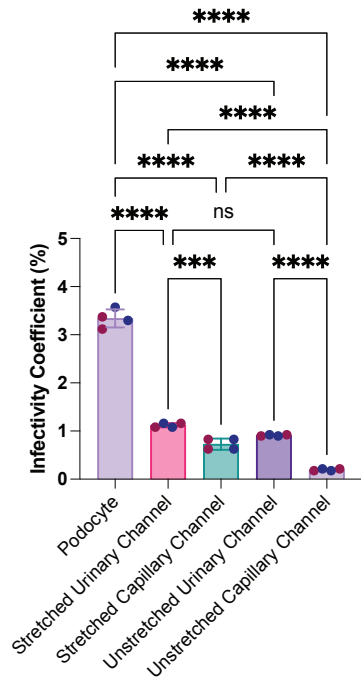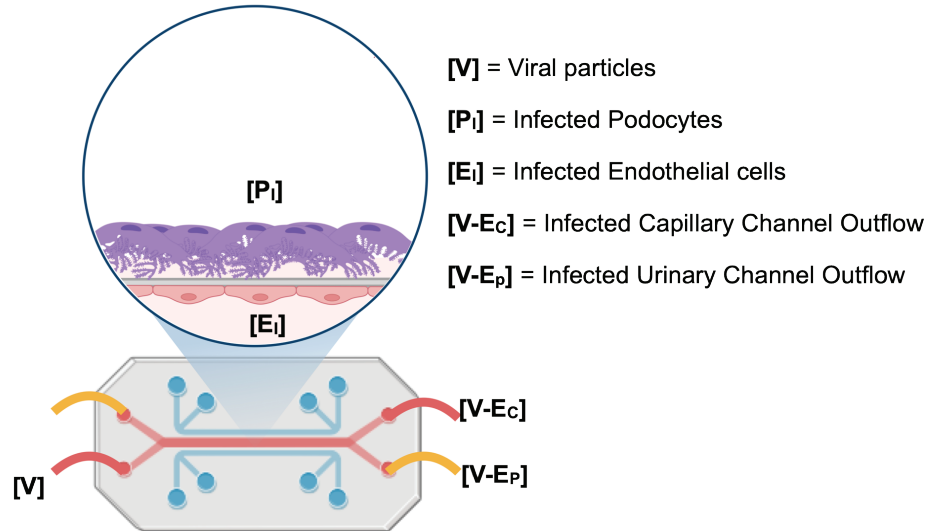

Supplement: Supplementary file 4 — Supplementary file4 (PDF 1765 KB) [file 12195_2025_851_MOESM4_ESM.pdf]

Figure 1D

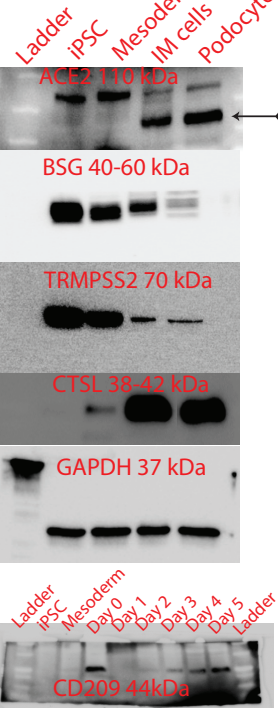

Figure 2B

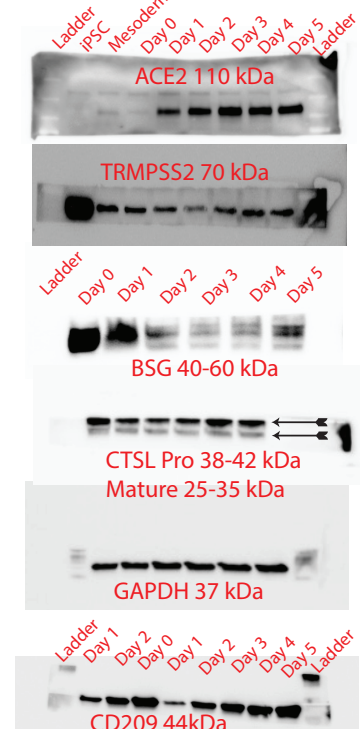

Figure 3E

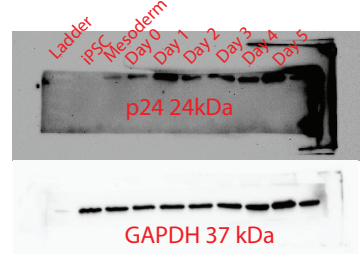

Figure 4B

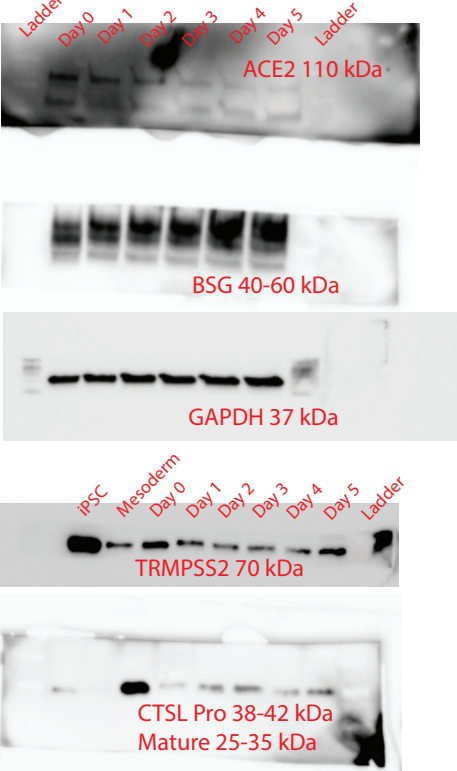

Figure 4C

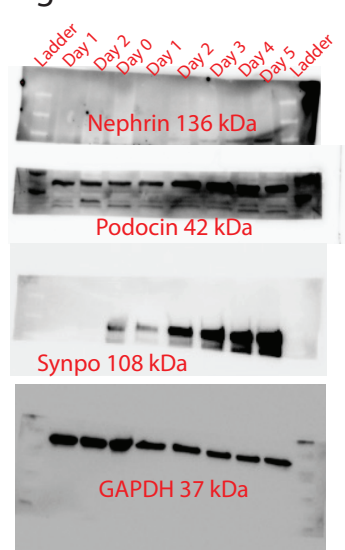

Figure 4D

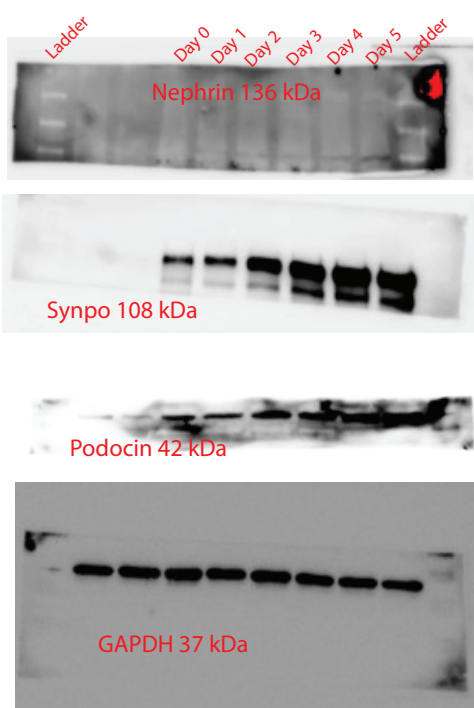

Supplement: Supplementary file 5 — Supplementary file5 (PDF 10123 KB) [file 12195_2025_851_MOESM5_ESM.pdf]

Human iPS cells

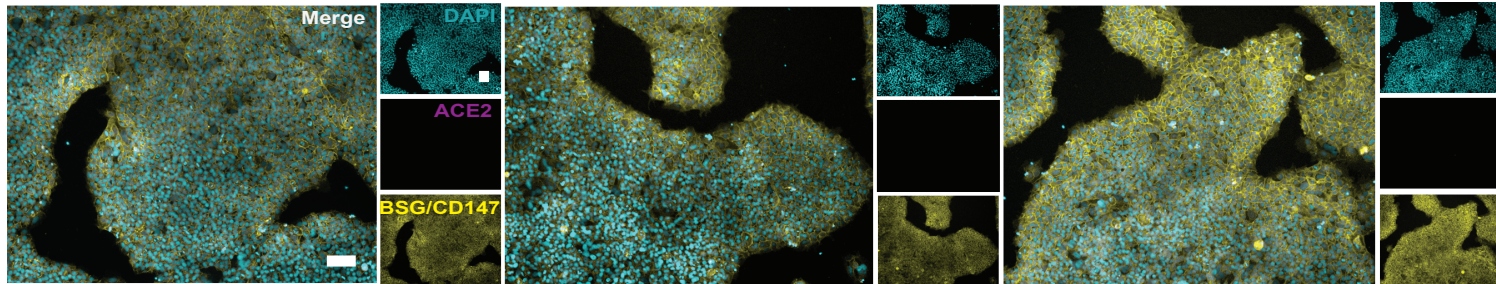

Mesoderm

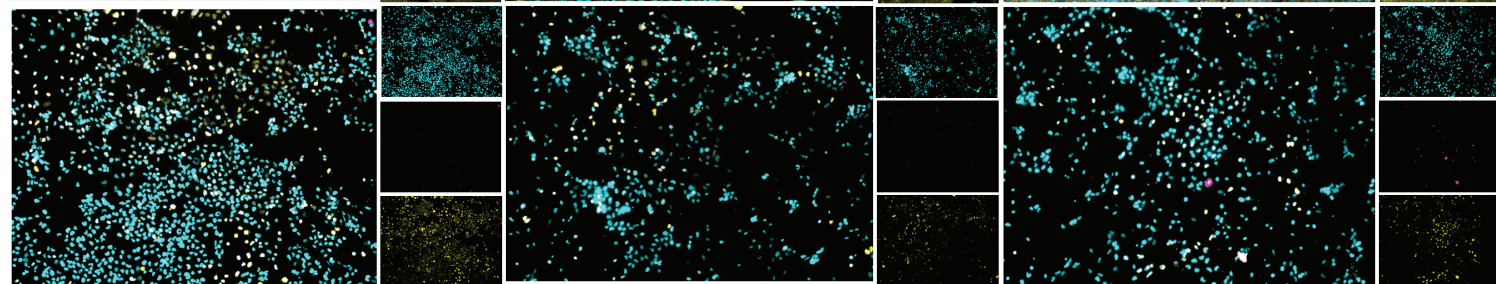

Intermediate Mesoderm

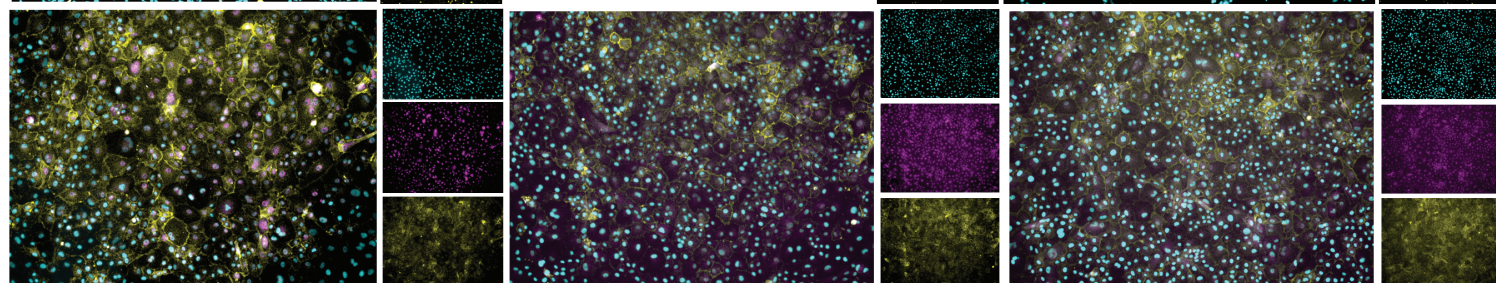

Podocyte

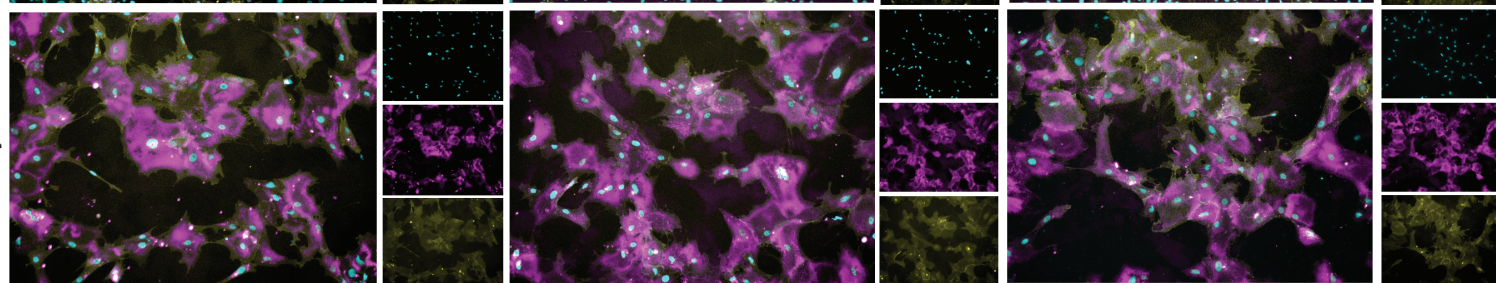

Supplement: Supplementary file 6 — Supplementary file6 (PDF 25820 KB) [file 12195_2025_851_MOESM6_ESM.pdf]

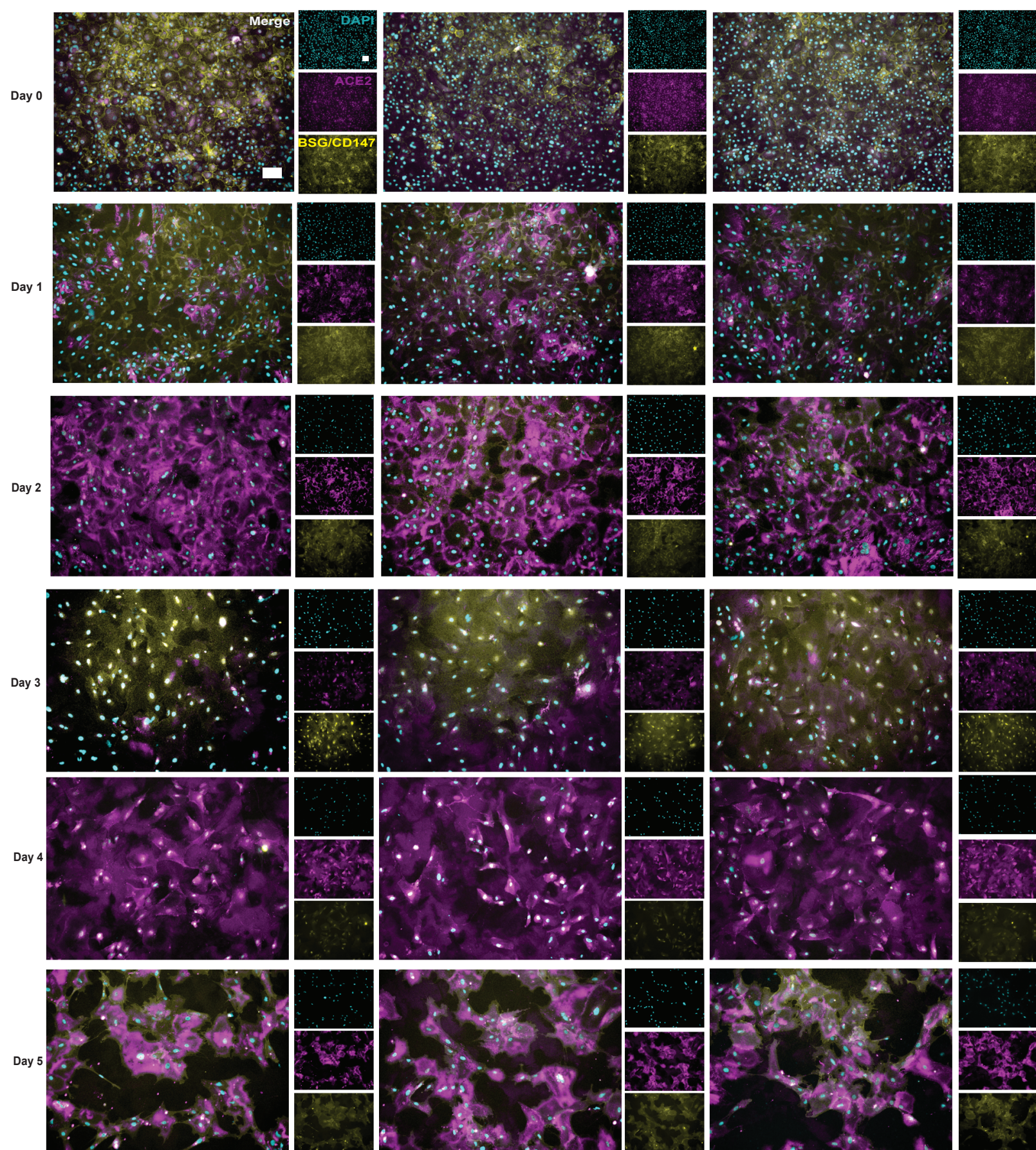

Supplement: Supplementary file 7 — Supplementary file7 (PDF 22075 KB) [file 12195_2025_851_MOESM7_ESM.pdf]

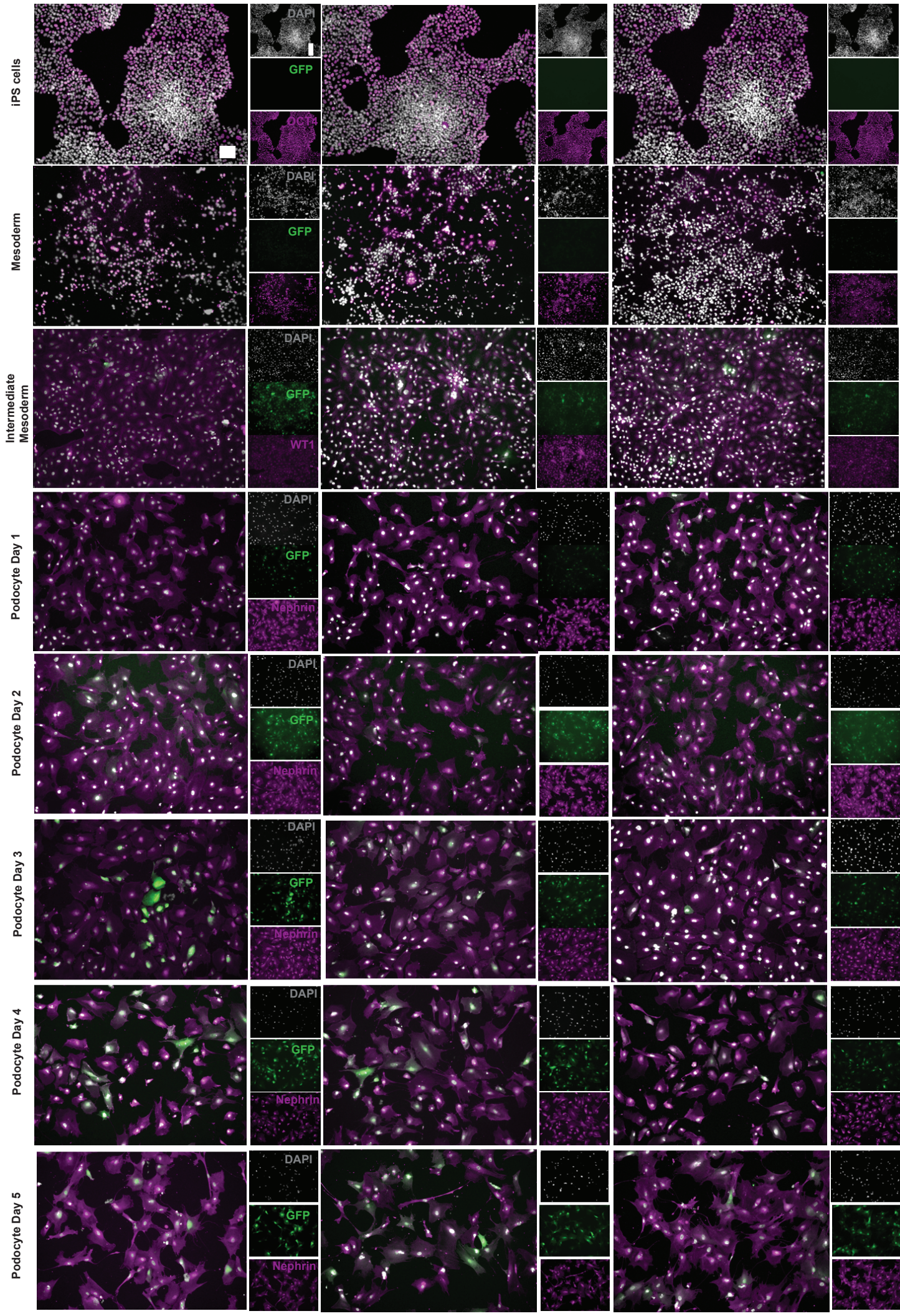

Supplement: Supplementary file 8 — Supplementary file8 (PDF 14977 KB) [file 12195_2025_851_MOESM8_ESM.pdf]

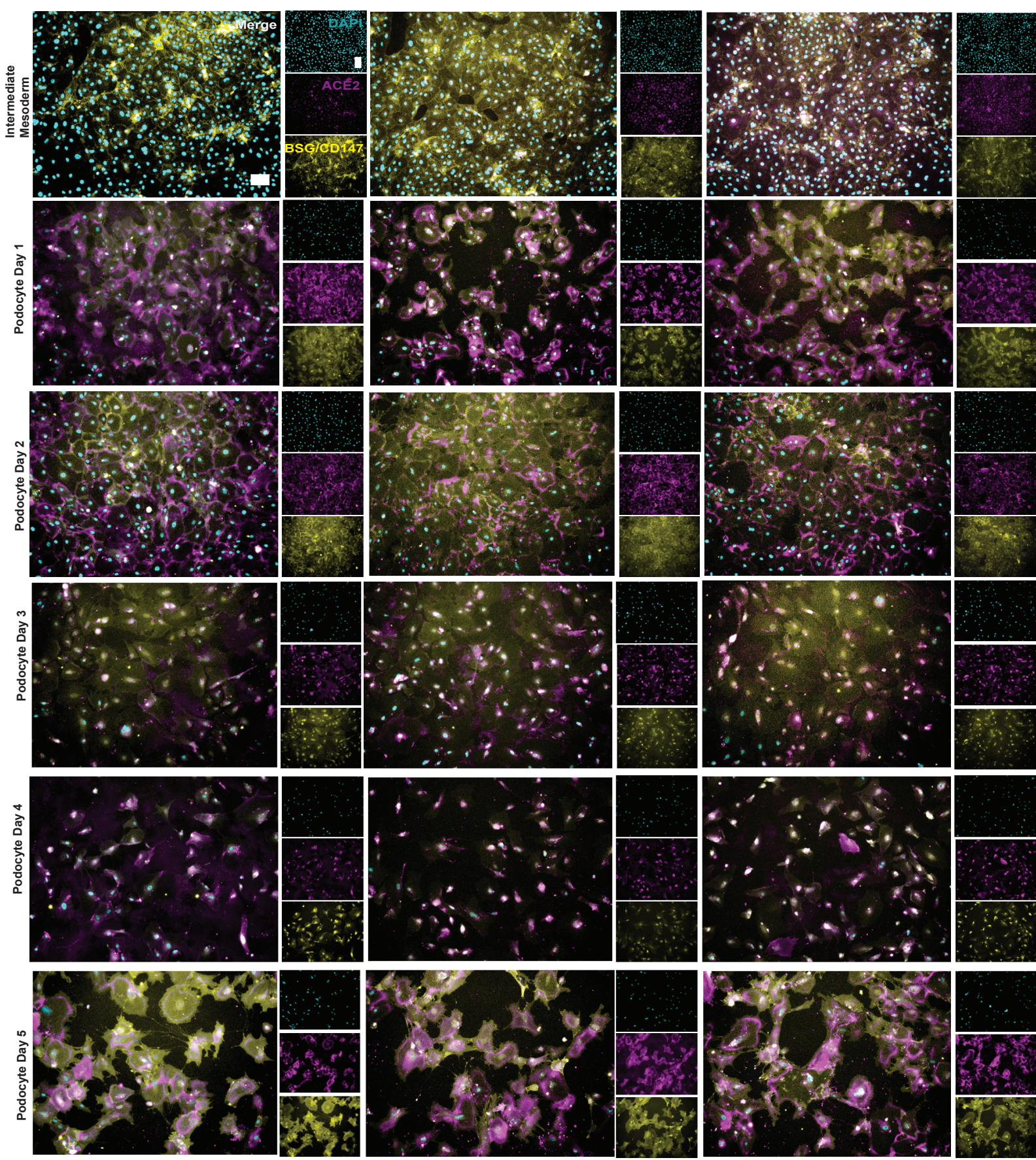

Supplement: Supplementary file 9 — Supplementary file9 (PDF 20501 KB) [file 12195_2025_851_MOESM9_ESM.pdf]
