# Supplementary material for: A Vascularized Human Organ Chip Reveals SARS-CoV-2 Susceptibility in Developmentally Guided Tissue Maturation
Source: Cell Mol Bioeng. 2025 Jul 22;18(5):453–71. doi: 10.1007/s12195-025-00851-4 (PMC12579647; doi:10.1007/s12195-025-00851-4)
Supplement: Supplementary file 10 — Supplementary file10 (DOCX 17 KB) [file 12195_2025_851_MOESM10_ESM.docx]

# **Supplementary Material**

Supplementary Table S1: Primer sequences used in the study.

Supplementary figures 1, 2, 3 and 4

Extended figures 1, 2, 3 and 4

**Supplementary & Extended Figure legends**

**Supplementary Figure 1: qPCR data of iPS cells, mesoderm, IM and podocytes and Morphological changes in developing and mature podocytes** from IM (Day 0) to the more specialized human iPS cell-derived podocyte stage (Day 5). (A) qPCR quantification of human spike-associated genes and the viral processing factors Transmembrane Serine Protease 2 (TMPRSS2) and cathepsin L (CTSL) in the cell lineages (normalized to human iPS cell group). SIGLEC9, **Sialic acid-binding Ig-like lectin 9;** CLEC10A, **C-type lectin domain family 10 member A; CD33, Myeloid cell surface antigen CD33; ACE2,** Angiotensin Converting Enzyme 2; BSG/CD147, Basigin/CD147 molecule; CD209, CD209 Antigen; MYO6, **Unconventional myosin-VI;** SIGLEC10, **Sialic acid-binding Ig-like lectin 10;** ACTR3, **Actin-related protein 3.** One-way analysis of variance (ANOVA) with Sidak’s multiple comparison test was used to determine statistical significance. Only p-values of 0.05 or lower were considered statistically significant. p > 0.05 [ns, not significant], p < 0.05 [*], p < 0.01 [**], p < 0.001 [***], p < 0.0001 [****]). Error bars indicate ± SEM (standard error of the mean). (B) Phase contrast images of human iPS cell-derived podocyte morphology from undissociated IM cells up to day 5 podocytes

**Supplementary Figure 2**: **Effect of S-pseudotyped virus on podocyte marker expression**. qRT-PCR analysis of (**A**) uninfected and (**B**) Infected cells measuring the expression levels of mature and progenitor markers (**C**) Phase contrast images showing morphology of human iPS cell-derived podocytes from undissociated IM cells (Day 0) to fully differentiated podocytes (Day 5). (**D**) qRT-PCR data evaluating changes in human iPS cell markers in infected and uninfected cells. (**E**) Phase contrast images of infected and uninfected iPS cells. (**F**) qRT-PCR data evaluating changes in mesoderm markers in infected and uninfected cells (**G**) Phase contrast images of infected and uninfected Mesoderm cells. Scale bars: 100 µm.

**Supplementary Figure 3**: **Infectivity of glomerulus chip to S-pseudotyped virus.**(**A**) qRT-PCR analysis using Lenti-X titration kit comparing 2D (plate podocytes culture) and 3D (stretched and unstretched glomerulus chips) revealing a significant increase in uptake of viral particles in 2D (direct exposure) versus 3D (vascular perfusion) cultures. (**B**) Schematic overview for the infection coefficient calculation for the glomerulus chip with S-pseudotyped virus. **[V]**= Total Viral particles introduced into the glomerulus chip; **[V-E_C_]**= Infected Capillary Channel Outflow; **[E_I_]**= Infected Endothelial cells; **T_E_**= Total signal from the infected capillary channel; **[V-E_p_]**= Infected Urinary Channel Outflow; **[P_I_]**= Infected Podocytes; **T_P_**= Total signal from the infected urinary channel (**C**) Infectivity coefficient (%) comparing 2D culture to both stretched and unstretched channels. One-way analysis of variance (ANOVA) with Sidak’s multiple comparison test was used to determine statistical significance. Only p-values of 0.05 or lower were considered statistically significant (p > 0.05 [ns, not significant], p < 0.05 [*], p < 0.01 [**], p < 0.001 [***], p < 0.0001 [****]). Error bars indicate ± SEM (standard error of the mean). Supplementary figure 3B was created with Biorender^TM^.

**Supplementary Figure 4:** Western blot images showing uncropped blots of all representative blots in the figures. Blots were cut for multiplexing purposes.

**Extended Figure 1:** Immunofluorescence images of figure 1D captured across independent biological replicates. ACE2 (magenta) and BSG/CD147 (yellow) expression in the different cell lineages counterstained with DAPI (cyan). Scale bar: 100 µm.

**Extended Figure 2:** Immunostaining images of ACE2 (magenta) and BSG/CD147 (yellow) expression in the cell types generated from developing podocytes and counterstained with DAPI (cyan). This figure shows additional images from the data described in figure 2E captured across multiple independent replicates. Scale bar: 100 µm.

**Extended Figure 3:** Immunofluorescent images showing GFP (green) and cell lineage identification markers for pseudotyped virus-infected cells. Respective lineage markers are shown in magenta; Oct4 (human iPS cells), Brachyury (Mesoderm), WT1 (IM cells), Nephrin (developing and mature podocytes). Cells were counterstained with DAPI (cyan). Scale bar: 100 µm. This figure provides additional immunofluorescent images of figure 3F captured for multiple independent experiments or biological replicates.

**Extended Figure 4:** Immunostaining images of ACE2 (magenta) and BSG/CD147 (yellow) expression in the infected cell types generated from podocyte induction. The cells were counterstained with DAPI (cyan). This figure provides additional images of figure 4H captured across multiple independent replicates. Scale bar: 100 µm.
